# The requirement for co-germinants during *Clostridium difficile* spore germination is influenced by mutations in *yabG* and *cspA*

**DOI:** 10.1371/journal.ppat.1007681

**Published:** 2019-04-03

**Authors:** Ritu Shrestha, Alicia M. Cochran, Joseph A. Sorg

**Affiliations:** Department of Biology, Texas A&M University, College Station, United States of America; University of Pittsburgh School of Medicine, UNITED STATES

## Abstract

*Clostridium difficile* spore germination is critical for the transmission of disease. *C*. *difficile* spores germinate in response to cholic acid derivatives, such as taurocholate (TA), and amino acids, such as glycine or alanine. Although the receptor with which bile acids are recognized (germinant receptor) is known, the amino acid co-germinant receptor has remained elusive. Here, we used EMS mutagenesis to generate mutants with altered requirements for the amino acid co-germinant, similar to the strategy we used previously to identify the bile acid germinant receptor, CspC. Surprisingly, we identified strains that do not require co-germinants, and the mutant spores germinated in response to TA alone. Upon sequencing these mutants, we identified different mutations in *yabG*. In *C*. *difficile*, *yabG* expression is required for the processing of key germination components to their mature forms (*e*.*g*., CspBA to CspB and CspA). A defined *yabG* mutant exacerbated the EMS mutant phenotype. Building upon this work, we found that small deletions in *cspA* resulted in spores that germinated in the presence of TA alone without the requirement of a co-germinant. *cspA* encodes a pseudoprotease that was previously shown to be important for incorporation of the CspC germinant receptor. Herein, our study builds upon the role of CspA during *C*. *difficile* spore germination by providing evidence that CspA is important for recognition of co-germinants during *C*. *difficile* spore germination. Our work suggests that two pseudoproteases (CspC and CspA) likely function as the *C*. *difficile* germinant receptors.

## Author summary

Germination by *C*. *difficile* spores is one of the very first steps in the pathogenesis of this organism. The transition from the metabolically dormant spore form to the actively-growing, toxin-producing vegetative form is initiated by certain host-derived bile acids and amino acid signals. Despite near universal conservation in endospore-forming bacteria of the Ger-type germinant receptors, *C*. *difficile* and related organisms do not encode these proteins. In prior work, we identified the *C*. *difficile* bile acid germinant receptor as the CspC pseudoprotease. In this manuscript, we implicate the CspA pseudoprotease as the *C*. *difficile* co-germinant receptor. *C*. *difficile cspA* is encoded as a translational fusion to *cspB*. The resulting CspBA protein is processed post-translationally by the YabG protease. Inactivation of *yabG* resulted in strains whose spores no longer responded to amino acids or divalent cations as co-germinants and germinated in response to bile acid alone. Building upon this, we found that small deletions in the *cspA* portion of *cspBA* resulted in spores that could germinate in response to bile acids alone. Our results suggest that two pseudoproteases regulate *C*. *difficile* spore germination and provide further evidence that *C*. *difficile* spore germination proceeds through a novel spore germination pathway.

## Introduction

*Clostridioides difficile* (formerly *Clostridium difficile*) [1–3] is a Gram-positive, spore-forming pathogenic bacterium, and has become a leading cause of nosocomial diarrhea in the United States [4, 5]. *C*. *difficile* infection (CDI) is commonly the result of disruption to the gut microflora caused by antibiotic use [5–7]. Due to the broad-spectrum nature of many antibiotics, alterations to the ecology of the colonic microbiome results in the loss of the colonization resistance that is provided by the microbiota. Subsequently, patients are treated with other, broad-spectrum, antibiotics (*e*.*g*., vancomycin or fidaxomicin) which treat the actively growing, toxin-producing, vegetative cells [8]. Although these antibiotics alleviate the primary symptoms of disease, the continued disruption to the colonic microbiome results in frequent CDI recurrence. The symptoms of CDI are caused by the actions of two secreted toxins. TcdA (an enterotoxin) and TcdB (a cytotoxin) are endocytosed by the colonic epithelium and inactivate the Rho-family of small GTPases leading to loss of barrier function and inflammation of the colonic epithelium [7].

Though *C*. *difficile* vegetative cells produce the toxins that cause CDI, they are strictly anaerobic and only survive short periods of time outside the anaerobic colonic environment [9]. However, the spores that are produced by the vegetative form are critical for transmission between hosts because of their resistance to environmental factors such as heat, UV, chemicals and, importantly, oxygen [10–14]. The overall architecture of spores is conserved among all endospore-forming bacteria, *C*. *difficile* included. The centrally-located core is composed of DNA, RNA, ribosomes and proteins necessary for the outgrowth of a vegetative cell, post germination [11, 14]. The DNA in the core is protected from UV damage by small acid soluble proteins (SASPs) and much of the water in the core is replaced by pyridine-2, 6-dicarboxylic acid (dipicolinic acid; DPA), chelated with calcium (CaDPA), which provides heat resistance to the spores [11, 14, 15]. The core is surrounded by an inner membrane composed of phospholipids with minimal permeability to small molecules, including water [15]. A thin germ-cell-wall layer surrounds the inner membrane and becomes the cell wall of the vegetative cell upon outgrowth. A thick layer of specialized peptidoglycan (cortex) surrounds the germ cell wall and helps constrain the core against osmolysis [15]. Finally, surrounding the cortex is the outer membrane which, initially, serves as an organization structure / point for the coat layers but may be lost later during spore germination [11, 16–20]. All these features of endospores contribute to ensuring that the spores remain metabolically dormant.

Though dormant, spores still sense their environment for species-specific germination-inducing small molecules and, when appropriate germinants are present, initiate the process of spore germination. Much of our knowledge of spore germination comes from studies in *Bacillus subtilis* (a model organism for studying spore formation and germination). In *B*. *subtilis*, and most other endospore-forming bacteria, germination is activated upon binding of the germinants to the Ger-type germinant receptors that are deposited in or on the inner spore membrane [21, 22]. In *B*. *subtilis*, this event triggers an irreversible process whereby CaDPA is released through a channel composed of the SpoVA proteins [23–29]. The release of CaDPA is an essential step for the germination process because it results in the rehydration of the spore core and permits the eventual resumption of metabolic activity. In *B*. *subtilis*, the cortex peptidoglycan layer is then degraded, and this event can be activated by the CaDPA that is released from the core [30].

In contrast to *B*. *subtilis*, *C*. *difficile* does not encode orthologues of the Ger-type germinant receptors suggesting that the initiation of *C*. *difficile* spore germination is fundamentally different than what occurs in most other studied organisms [10]. Germination by *C*. *difficile* spores is triggered in response to certain bile acids in combination with certain amino acids [10, 31, 32]. In all identified *C*. *difficile* isolates, the cholic acid-derivative, taurocholate (TA), is the most efficient bile acid at promoting spore germination, and glycine is the most efficient amino acid co-germinant [33–35]. Recently, calcium was identified as an important contributor to spore germination and may synergize with other co-germinants to enhance *C*. *difficile* spore germination [36, 37].

In a screen to identify ethyl methane sulfonate (EMS)-generated mutants that do not respond to TA as a spore germinant, we identified the germination-specific, subtilisin-like pseudoprotease, CspC, as the *C*. *difficile* bile acid germinant receptor [38]. Prior to work performed in *C*. *difficile*, the Csp locus was best studied in *Clostridium perfringens* [39–42]. *C*. *perfringens* encodes three Csp proteases, CspB, CspA, and CspC, that are predicted to cleave the inhibitory pro-peptide from proSleC, a spore cortex hydrolase that degrades the cortex peptidoglycan, thereby activating the protein [39–42]. In *C*. *difficile*, CspB and CspA are encoded by one ORF, *cspBA*, and the resulting protein is post-translationally autoprocessed by CspB into CspB and CspA and then further processed by the sporulation specific protease, YabG (CspA and CspC do not undergo autoprocessing) [14, 43–45]. CspC is encoded downstream of *cspBA* and is part of the same transcriptional unit. Interestingly, the catalytic residues in CspA and CspC are mutated, rendering these proteins catalytically inactive suggesting that only the CspB protein can process proSleC to its active, cortex degrading form [38, 45]. Although present in the spore, and essential for *C*. *difficile* spore germination, the CspA pseudoprotease has only been shown to regulate the incorporation of CspC into the spore [43, 44].

In our working model for spore germination, activation of CspC by TA leads to the activation of the CspB protein which cleaves proSleC into its active form. Activated SleC degrades cortex and the core releases CaDPA in exchange for water by a mechanosensing mechanism [46]. Because the receptor with which amino acids interact is unknown, we sought to screen for chemically-generated mutants that have altered amino acid requirements during spore germination (similar to the strategy used to identify the bile acid germinant receptor [38]). Here, we report that a mutation in the *yabG* gene results in strains whose spores no longer respond to or require co-germinants but respond to TA alone as a spore germinant. We hypothesize that the misprocessing of CspBA in the *yabG* mutant leads to this phenotype and provide evidence that short deletions in CspA alter the requirements for a co-germinant during spore germination. Our results implicate CspA in the recognition of amino acid and divalent cation co-germinants.

## Materials and methods

### Growth conditions

*C*. *difficile* strains were grown on BHIS agar medium (Brain heart infusion supplemented with 5 g / L yeast extract and 1 g / L L-cysteine) in an anaerobic chamber (Model B, Coy Laboratories Grass Lake, MI) at 37°C (85% N_2_, 10% H_2_, and 5% CO_2_). Antibiotics were added as needed (15 μg / mL of thiamphenicol, 10 μg / mL lincomycin, and 20 μg / mL uracil). Deletion mutants were selected on *C*. *difficile* minimal medium (CDMM) supplemented with 5 μg / mL 5-fluoroorotic acid (FOA) and 20 μg / mL uracil [47]. *Bacillus subtilis* was used as a conjugal donor strain to transfer plasmids into *C*. *difficile* and was grown on LB medium with 5 μg / mL of tetracycline and 2.5 μg / mL of chloramphenicol. Conjugation was performed on TY medium (3% Bacto Tryptone, 2% yeast extract, and 0.1% thioglycolate) with or without uracil. *E*. *coli* DH5α, *E*. *coli* BL21(DE3) and *E*. *coli* MB3436 were grown on LB medium supplemented with 20 μg / mL chloramphenicol and / or 100 μg / mL ampicillin at 37°C. All strains are listed in Table 1.

**Table 1:** Strain list.

*C*. *difficile* Strain	Description	Plasmids used	Reference
UK1	Wild type, ribotype 027		[69]
R20291	Wild type, ribotype 027		[70]
CRG2359	R20291 Δ*pyrE*		[47]
RS08	*yabG*::*ermB* in R20291 background	pRS93	This study
RS10	*sleC*::*ermB* in R20291 background	pCA6	This study
RS19	CRG2359 with restored *pyrE*	pRS107	This study
RS16	Δ*cspBAC* CRG2359 with restored *pyrE*	pRS105, pRS107	This study
RS20	CRG2359 with Δ522–548 deletion in CspB	pRS117	This study
RS21	CRG2359 with Δ560–586 deletion in CspA	pRS116	This study
RS23	CRG2359 with Δ542–567 in CspBA	pRS114	This study
RS24	CRG2359 with Δ537–571 in CspBA	pRS115	This study
RS26	RS21 with restored *pyrE*	pRS107	This study
RS27	CRG2359 with Δ560–610 in CspA	pRS118	This study
RS29	ΔSRQS (Δ580–584) in CspA	pRS120	This study
RS30	ΔSRQS (Δ117–121) in preproSleC	pRS121	This study
RS31	ΔSRQS in CspA and preproSleC	pRS120, pRS121	This study
**Other Strains**			
*E*. *coli* DH5α	Cloning vector		[71]
*E*. *coli* BL21(DE3)	Expression vector		[72]
*E*.*coli* Rosetta BL21(DE3)	Expression vector; pLysSRARE 2	pAC28, pAC35, pAC41, pAC42, pAC43	Novagen
*E*. *coli* MB3436	*recA*^+^ *E*. *coli* strain		Gift from Dr. Michael Benedik
*B*. *subtilis* Bs49	Tn916 donor strain, Tet^R^		[73]

### Spore purification

*C*. *difficile* spores were purified as described previously [32, 33, 46, 48]. Briefly, the strains without plasmids were grown on BHIS agar medium while strains with plasmid were grown on BHIS agar medium supplemented with 5 μg / mL thiamphenicol and allowed to grow for 4 days. Cells from each plate were scraped into 1 mL sterile water and incubated at 4°C overnight. Next, the cells were washed five times with water and combined into 2 mL total volume. The washed spores were layered on top of 8 mL of 60% sucrose and centrifuged at 4,000 x g for 20 minutes. The supernatant was discarded, and the spores were washed five more times with water and incubated at 4°C until use.

### EMS mutagenesis and phenotype enrichment

**Fig 1:**
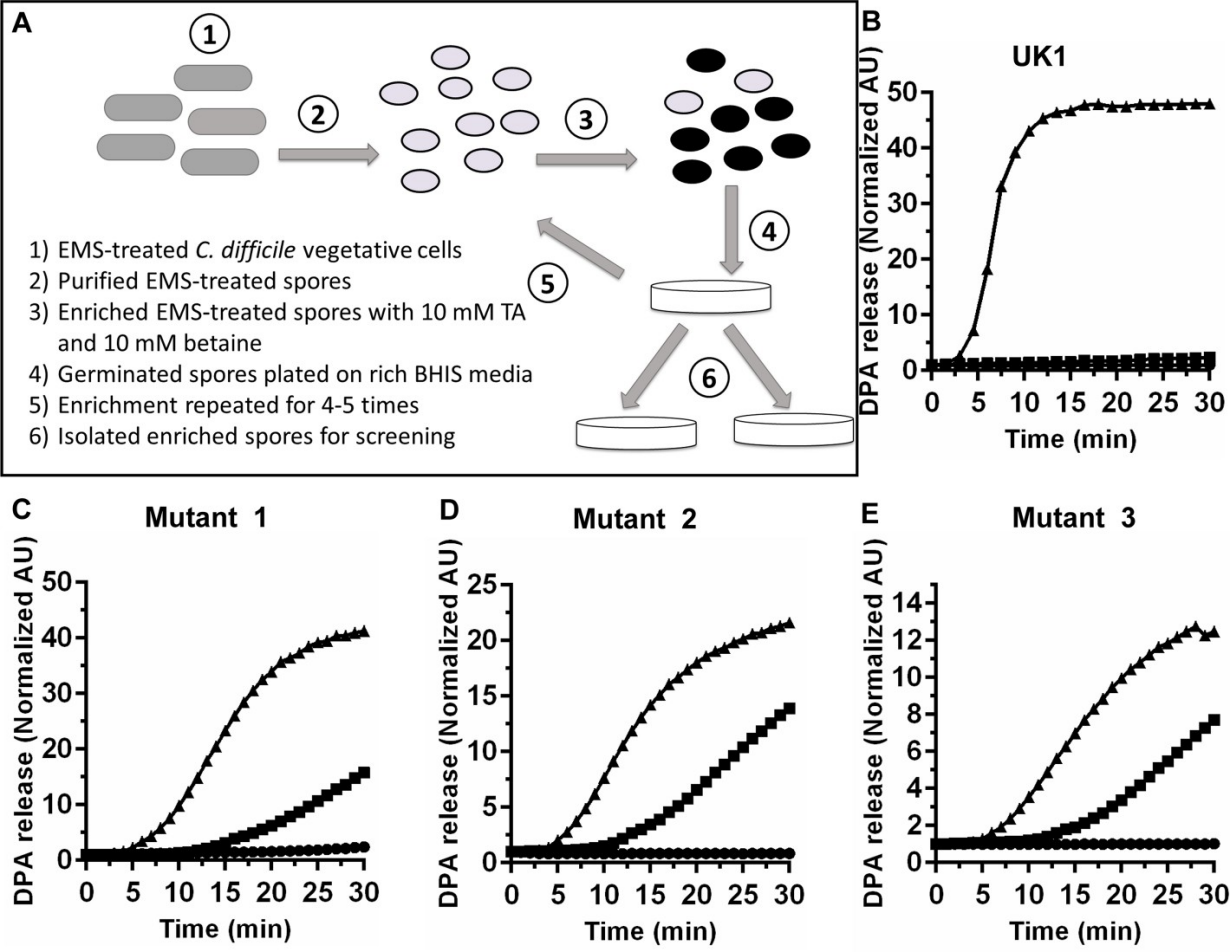
EMS mutagenesis generates *C*. *difficile* strains with altered requirements for the amino acid co-germinant. (A) Schematic of the EMS mutagenesis strategy where (1) *C*. *difficile* UK1 vegetative cells were treated with EMS, (2) EMS mutagenized cells were recovered and allowed to form spores, (3) purified, phase-bright spores (represented by gray ellipsoids) were suspended for 15 minutes in germination buffer supplemented with 10 mM TA and 10 mM betaine and then washed twice with buffer alone, (4) germinated, phase-dark spores (represented by dark ellipsoids) were plated onto BHIS agar medium to permit colony formation, (5) strains were enriched by exposure to TA and betaine, iteratively, 4–5 times, and (6) spores from the mutant strains were isolated for screening. The germination phenotype of wild type *C*. *difficile* UK1 (B) and mutant spores (C–E) were screened by measuring CaDPA release in presence of (black circle) 30 mM glycine, (black square) 10 mM TA or (black triangle) 10 mM TA and 30 mM glycine. The data points represent the data from a single germination experiment. Germination plots performed in triplicate yielded error bars that obscure the data. For transparency, the remaining germination plots can be found in S1 Fig.

An overnight culture of wild type *C*. *difficile* UK1 vegetative cells was diluted to an OD_600_ of 0.05 into two, separate 15 mL falcon tubes containing BHIS liquid and grown for 3–4 hrs. To one of the cultures, ethyl methane sulfonate (EMS) was added to a final concentration of 50 μg / mL; the other culture was untreated for use as a negative control. The cultures were grown for 3 hours and then centrifuged at 3,000 x g for 10 min. The supernatants were discarded, and pellets were washed two more times with BHIS. After the final wash, the pellets were suspended in 40 mL BHIS medium and allowed to recover overnight. The recovered EMS-treated cells were then plated onto 10–12 BHIS plates (25 μL on each plate) to produce spores. Spores were purified from the EMS-treated strain as described above. Purified spores were heat activated at 65°C for 30 minutes before enrichment as shown in Fig 1A. The EMS-treated spores were treated for 15 minutes with HEPES buffer (50 mM HEPES, 100 mM NaCl, pH 7.5) supplemented with 10 mM TA and 10 mM betaine and then washed twice with buffer. Germinated, washed spores were plated onto BHIS agar medium for spore formation. This procedure was repeated iteratively for 4–5 times before isolating candidate strains for phenotypic screening.

### Characterizing mutant phenotypes

The mutant spores were initially characterized by measuring the CaDPA release from germinating spores. Spores were heat activated at 65°C for 30 minutes and suspended in water at an OD_600_ = 50. The spores were then added to final OD of 0.5 in 100 μL final volume of HEPES buffer containing 250 μM Tb^3+^, 10 mM TA and / or 10 mM betaine in a 96 well plate. The CaDPA release was measured using a SpectraMax M3 plate reader for 30 minutes at 37°C with excitation at 270 nM and emission at 545 nM with a 420 nM cutoff.

The phenotype of the *yabG* mutant (RS08) and *cspBA* deletion mutants were determined by measuring changes to the OD_600_ and release of DPA. OD_600_ was monitored at 37°C for 1–2 hrs. using a plate reader. The spores were added to a final OD of 0.5 in HEPES buffer supplemented with 30 mM glycine alone or 10 mM TA alone or 10 mM TA and 30 mM glycine in 100 μL final volume. CaDPA release was measured as described above with a spore solution at a final OD_600_ of 0.25.

### DNA sequencing

High-quality genomic DNA was purified from logarithmically growing *C*. *difficile* cells [49]. Genomic DNA from the EMS-mutant strains and the wild type parent was sent for paired end sequencing at Tufts University Genomics Core. Reads were aligned to the R20291 genome using DNASTAR software and SNPs determined (DNASTAR, Madison, WI). RAW sequence (fastq) reads were uploaded to the NCBI Sequence Read Archive as follows (Table 2): *C*. *difficile* UK1 parent (SRS3677310), Mutant 20C (SRS3677309), Mutant 27E (SRS3677307), Mutant 30A (SRS3677308), Mutant 30C (SRS3677311), Mutant 31D (SRS3677312).

**Table 2:** Location of the mutations in *yabG* found in the EMS mutant strains.

Mutant	Reference	Coding region	Promoter region
20C (Mutant1)	4048886	G-T(Ala46Asp)	
27E (Mutant 2)	4049063		G-42A
30A (Mutant 3)	4048565	G-A (Pro153Leu)	
30C	4048913	C–T (Gly37Glu)	
31D	4049030		C-8T

Nucleotide positions in the promoter region are mapped from the ATG of the open reading frame (the *yabG* transcriptional start site is unknown)

### Molecular biology

The oligonucleotides used for making the strains and plasmids used in this study are listed in S1 Table. *yabG* and *sleC* mutants were created in the *C*. *difficile* R20291 background by using the TargeTron mutagenesis system. The potential insertion sites for targeting the group II intron were found using the Targetronics algorithm (Targetronics, LLC.) and a gBlock (Integrated DNA Technologies, San Jose, CA) of the group II intron targeted to *yabG* at the 279^th^ nucleotide, relative to the start codon, was ordered (S1 Table) and cloned into pJS107 at the HinDIII and BsrGI restriction sites using Gibson assembly. The ligation was then transformed into *E*. *coli* DH5α. The site to engineer a TargeTron insertion in *sleC* (pCA6) was previously described for *C*. *difficile* UK1 [48] and the same protocol was used to engineer the insertion into R20291. The TargeTron insertion plasmids were isolated from *E*. *coli* DH5α and transformed into *E*. *coli* MB3436 (a *recA*^+^ strain) and then into *B*. *subtilis* BS49. Conjugation between *B*. *subtilis* and *C*. *difficile* R20291 was performed on TY-agar medium for 24 hrs. before plating onto selection plates. Once the plasmid was inserted into *C*. *difficile*, the colonies were screened for tetracycline-sensitive and thiamphenicol-resistant colonies and confirmed with PCR. *yabG* or *sleC* TargeTron mutants were selected by plating the colonies onto BHIS supplemented with lincomycin. Lincomycin-resistant colonies were tested by PCR and confirmed by sequencing the mutation. *yabG* and *sleC* TargeTron mutants were renamed as RS08 and RS10, respectively (Table 1).

A *yabG* complementing plasmid (pRS97) was created using primers 5' XbaI_Prom_YabG and 3' YabG_XhoI to amplify the *yabG* promoter and *yabG* coding regions and cloned into pJS116. The complementing plasmid was then inserted into *C*. *difficile* RS08 by conjugation with *B*. *subtilis*, as described above.

All recombinant proteins were expressed from the pET22b plasmid. For expression of the indicated *yabG* and *sleC* alleles, the indicated alleles were amplified from the *C*. *difficile* R20291 background. Plasmids were created by Gibson assembly of the amplified fragments into pET22b using either NdeI and BamHI (pAC28, pAC35, pAC43, and pAC42) or NdeI and XhoI (pKS08 and pAC41) restriction sites, then transformed into *E*.*coli* DH5ɑ for isolation and subsequently into *E*.*coli Rosetta* BL21 pLysSRare for protein expression. pAC28 and pAC35 were constructed using oligonucleotides pet22b_YabG_Fp and pet22b_YabG_Rp and used either *C*. *difficile* R20291 or Mutant 1 (Table 2) as template. pAC41 was constructed using oligonucleotides 5’pET_SleC and 3’pET_SleC using *C*. *difficile* RS31 as template. pAC43 was constructed using oligonucleotides pet22b_SleC_Fp with Pet22b_SleC_QSELI DEL_Rp and Pet22b_SleC_QSELI DEL_Fp with pet22b_yabg_SleC6his_Rp. Finally, pAC42 was constructed with oligonucleotides pet22b_YabG_Fp with YABG_slec_Rp and yabg_SLEC_Fp with pet22b_yabg_SleC6his_Rp.

To engineer the required site-specific deletions, the *pyrE*-mediated allelic exchange strategy was used with the *C*. *difficile* CRG2359 strain (R20291 Δ*pyrE*) [47]. Briefly, 1 kb upstream and 1 kb downstream fragments that surround the desired mutation in *cspBA* or *preprosleC* were cloned into pJS165 using primers listed in S1 Table. The plasmids were inserted into *C*. *difficile* CRG2359 strain using *B*. *subtilis* conjugation as described above. The strains containing the plasmids were then passaged several times to encourage the formation of single recombinants before passing onto CDMM-FOA-uracil medium. Thiamphenicol-sensitive candidate strains were tested by PCR for the desired mutations and confirmed by sequencing for the mutagenized regions. Where indicated, *pyrE* was restored to wild type using pRS107 (Table 3).

**Table 3:** Plasmid list with primer pairs to make the plasmids.

Plasmid	Description	Oligonucleotides used (Supplemental Table 1)	Reference
pRS92	*yabG* G-block cloned into Zero blunt	813	This study
pRS93	Plasmid to make TT insertion in *yabG*		This study
pRS97	*yabG* Complementing plasmid	906/907, 924/925	This study
pCA6	Plasmid to make TT insertion in *sleC*		[48]
pMTLYN4	Plasmid to make deletion in *C*. *difficile* using allelic exchange		[47]
pJS107	TargeTron vector		[38]
pJS116	Empty vector		[38]
pJS165	Tn916Ori cloned into pMTLYN4 to use *B*. *subtilis* conjugation for gene insertion	207, 208	This study
pRS110	pJS116 with Δ542–567 in CspBA	578/1232, 1233/1056	This study
pRS111	pJS116 with Δ537–571 in CspBA	578/1234, 1235/1056	This study
pRS112	pJS116 with Δ560–586 in CspBA	578/1236, 1237/1056	This study
pRS113	pJS116 with Δ522–548 in CspBA	578/1238, 1239/1056	This study
pRS114	pJS165 with 1Kb upstream and 1Kb downstream od Δ542–567 in CspBA	1160, 1163	This study
pRS115	Plasmid to make Δ537–571 in CspBA cloned into pJS165	1160, 1163	This study
pRS116	Plasmid to make Δ560–586 in CspBA cloned into pJS165	1160, 1163	This study
pRS117	Plasmid to make Δ522–548 in CspBA cloned into pJS165	1160, 1163	This study
pMTLYN2C	Plasmid to restore *pyrE*		[47]
pRS107	Tn916Ori cloned into pMTLYN2C to use *Bacillus subtilis* for restoring pyrE	207, 208	This study
pRS118	Plasmid to make Δ560–610 deletion in CspA	1160/1287, 1163/1288	This study
pRS120	Plasmid to make SRQS deletion in CspA	1160/1329, 1163/1330	This study
pRS121	Plasmid to make SRQS deletion in preproSleC	1361/1362, 1363/1364	This study
pRS105	Plasmid to make the *cspBAC* deletion in CRG2359	943, 1086,1087,1088	This study
pKS08	*preproSleC* expression plasmid	466/467	[33]
pAC28	*yabG*_*6HIS*_ expression plasmid	1466/1467	This study
pAC35	*yabG*_*A46D/6HIS*_ expression plasmid	1466/1467	This study
pAC41	*preprosleC*_*ΔSRQS*_ expression plasmid	466/467	This study
pAC42	*yabG*–*preprosleC* co-expression plasmid	1466/1572, 1573/1680	This study
pAC43	*preproSleC*_*ΔQSELI*_ expression plasmid	1681/1682, 1683/1680	This study

### Western blot

Samples were prepared for CspB, CspC, and SleC western blot by extracting soluble proteins from 2 x 10^9^ / mL spores [R20291, *yabG*::*ermB*, *yabG*::*ermB* pRS97 (p*yabG*)]. For the protein standard, recombinant CspB, SleC and CspC proteins were purified using a previously described protocol [33]. Standard amount of protein or number of spores were solubilized in NuPAGE sample buffer (Life Technologies) and heated at 95°C for 20 minutes. Equal volume of spore extracts and recombinant CspB, CspC or SleC standard proteins were separated by SDS-PAGE. Proteins were then transferred onto low-fluorescence polyvinylidene difluoride membrane (PVDF) at 30V for 16 hours. The membranes were then blocked in 10% skimmed milk in TBS (Tris-buffered saline) and washed thrice with TBS containing 0.1% (vol / vol) Tween-20 (TSBT) for 20 minutes each at room temperature. The membranes were then incubated with anti-CspB, anti-CspC or anti-SleC antibodies for 2 hours and washed with TSBT thrice. For the secondary antibody, AlexaFluor 555-labeled donkey anti-rabbit antibody was used to label the membranes for 2 hours, in the dark. The membranes were washed again, thrice, with TBST, in the dark, and scanned with GE Typhoon Scanner using Cy3 setting, an appropriate wavelength for the Alexa Flour 555 fluorophore. The fluorescent bands were quantified using ImageQuant TL 7.0 image analysis software. Intensity of the extracted protein in each blot was compared to the standard curve that was generated from the recombinant protein included on each blot.

To analyze SleC activation, equal number of spores were suspended in HEPES buffer supplemented with 30 mM glycine or 10 mM TA or 10 mM TA and 30 mM glycine and incubated at 37°C for 1 hr. to 2 hrs. (aerobically). The samples were then centrifuged at 15,000 X g for 1 minute and pellets were suspended in NuPAGE sample buffer and heated for 20 min at 95°C. The suspension was centrifuged at max rpm for 10 min. The supernatant was separated and transferred into new tubes. The samples were stored at -20°C until use. For CspC, CspA and CspB western blots, an equal number of spores were suspended in HEPES buffer and boiled in NuPAGE buffer to extract the protein and loaded in 10% SDS PAGE gel. The spore extracts were then transferred onto nitrocellulose membrane for western blot analysis.

### Protein expression and purification

Plasmids (pAC41, pAC42, pAC43) were transformed into *Rosetta* strain *E*.*coli* [pKS08 was transformed into BL21(DE3)] and incubated overnight at 37°C on LB agar plates containing chloramphenicol and ampicillin. Plates were scraped into 1 mL LB and used to inoculate 1 L of 2XTY medium supplemented with chloramphenicol and ampicillin in baffled flasks, such that the starting culture OD_600_ = 0.01. Cultures were incubated at 37°C until the OD_600_ measured between 0.6 and 0.7, at which point they were induced with 250 μL of 1 M IPTG then returned to the incubator for an additional overnight (12–16 hours) incubation at 16°C. Cultures were pelleted by centrifugation at 4°C for 30 min at ~6,000 x g. The supernatant was discarded and the culture pellets were frozen at -80°C until use. The cells were lysed in 300 mM NaCl, 50 mM Tris-HCl, pH 7.5, 15 mM imidazole, and 10% glycerol. One liter of culture pellet was resuspended in 25 mL of lysis buffer supplemented with PMSF, lysozyme and DNase I and rocked on ice for 30 min prior to sonication. Samples were then centrifuged for 30 min at 6,000 x g, 4°C and the supernatant was combined with 1 mL of Ni-NTA beads. Samples were rocked overnight at 4°C. Beads were washed twice with 300 mM NaCl, 50 mM Tris-HCl, pH 7.5, 30 mM imidazole, and 10% glycerol and then eluted in the same buffer but supplemented with 500 mM imidazole. Samples were concentrated to <1 mL using a 10K MWCO centrifugal device and the recombinant protein further purified by FPLC, after which they were again concentrated.

Plasmids containing wildtype (pAC28) or mutant (pAC35) *yabG* were expressed slightly differently for the SleC-YabG incubations. Plasmids were also transformed into the *Rosetta E*. *coli* strain, and then cultured at a starting OD_600_ = 0.01 in a 50 ml volume of LB-CM/AMP until OD_600_ reached between 0.6 to 0.7 at which point they were induced with 12.5 μL of 1 M IPTG then returned to the incubator for an additional hour at 37°C. YabG cultures were then pelleted by centrifugation at 6,000 x g at 4°C for 30 min. Supernatant was discarded and pellets were resuspended in 4 mL lysis buffer and sonicated. This bacterial lysate was immediately used in the incubation with the indicated, recombinantly expressed and purified, SleC alleles.

### Statistical analysis

All germination assays were performed in technical triplicate of biological duplicates and data points represent the averages from these data sets. Error bars represent the standard error of the mean. A 1-way ANOVA with Tukey’s multiple comparisons test was used to compare the quantified protein amounts. For quantification of proteins, each blot was loaded with 5 standard proteins and three spore samples.

## Results

### Identifying *C*. *difficile* mutants with altered co-germinant requirements

In order to identify the receptor with which amino acid co-germinants interact, we used a strategy that was previously used to identify the bile acid germinant receptor (CspC) [38]. Although other strategies, such as Tn-seq, could be used to generate random mutations, most of these will result in germination null phenotypes and do not permit the screening of subtler phenotypes. As shown in Fig 1A, wild type *C*. *difficile* UK1 vegetative cells were exposed to EMS and recovered. Purified spores derived from the mutagenized bacteria were then germinated in buffer supplemented with 10 mM TA and 10 mM betaine. The structural difference between glycine and betaine is the presence of three methyl groups attached to the N-terminus rather than two hydrogen atoms. Because betaine is a glycine analog and does not stimulate spore germination when added with TA [50], we hypothesized that we could isolate change-of-function mutants that recognize betaine as a germinant or those that no longer require glycine as a co-germinant. Spores incubated in the presence of buffered TA and betaine were then plated and allowed to form spores. Potential mutants were enriched with this strategy 4–5 times before isolating colonies and screening for phenotypes. Across several mutagenesis experiments, the most commonly-observed phenotypes were strains that did not require the co-germinant glycine to germinate and germinated in response to taurocholate only (TA-only). As shown in Fig 1B, wild type *C*. *difficile* UK1 spores required both TA and glycine to stimulate the release of CaDPA from the core. However, spores purified from isolates derived from separate EMS mutageneses released CaDPA in the presence of TA only (Fig 1C, 1D and 1E). Importantly, though, these mutants still responded to glycine, the germination efficiency / rate increased upon glycine addition. However, none of the mutants recognized betaine as a co-germinant. To identify the mutation(s) that caused this phenotype, five different mutant strains (isolated from 4 independent EMS mutageneses) and a wild-type control were sent for genome re-sequencing. Surprisingly, when the sequences of the mutant strains were compared to the sequence of the wild-type parent, we identified SNPs common to all 5 mutants in the coding region or the promoter region of *yabG*, coding for a sporulation-specific protease (Table 2).

### Characterizing the function of *yabG*

**Fig 2:**
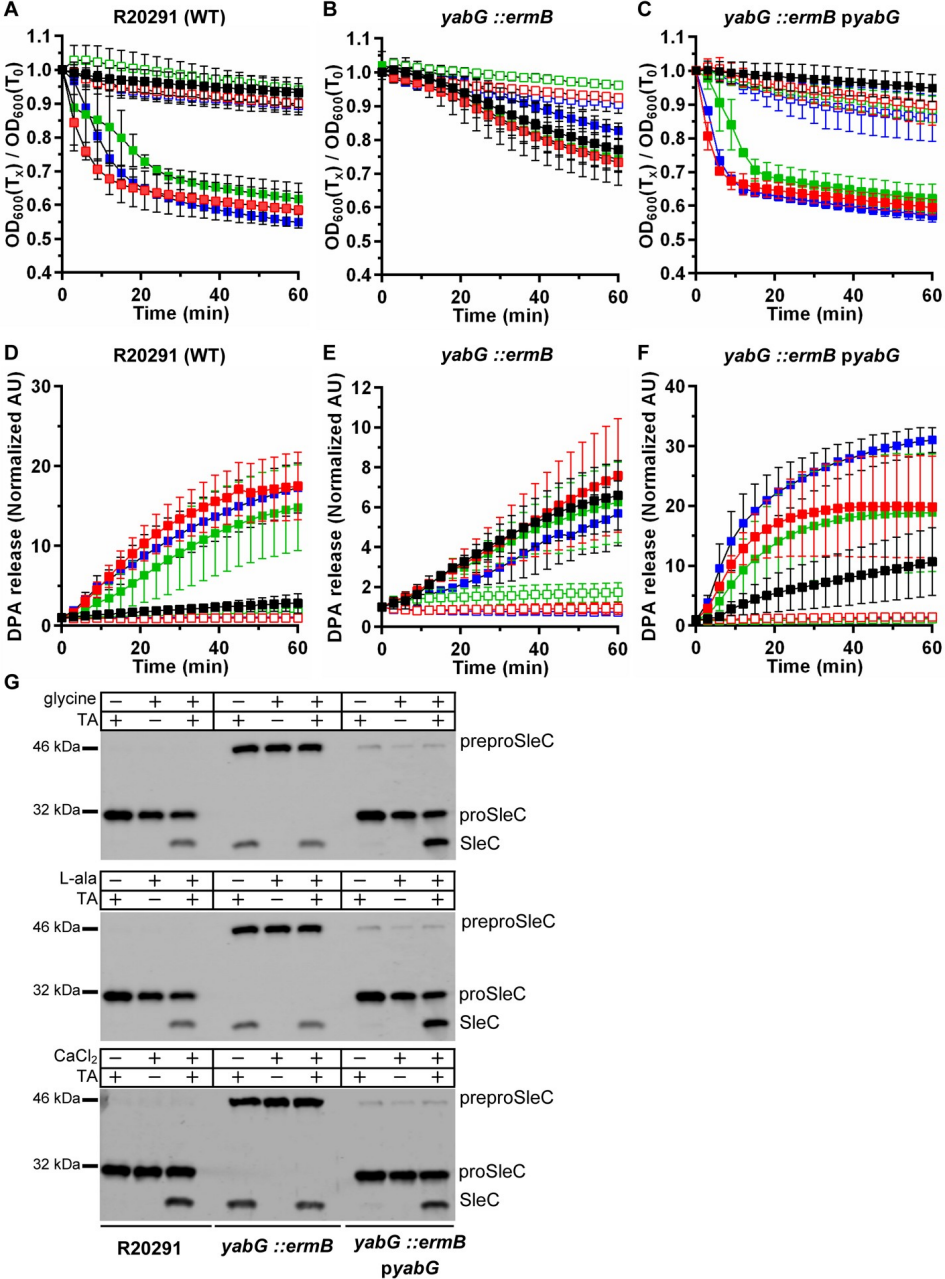
A mutation in *C*. *difficile yabG* results in spores that do not respond to co-germinants. Spores derived from *C*. *difficile* R20291, RS08 (*yabG*::*ermB*) and RS08 pRS97 (p*yabG*) strains were suspended in buffer supplemented 10 mM TA alone (black square), with 30 mM co-germinant alone (open symbols), or with TA and co-germinant (filled symbols); glycine (red), L-alanine (green), CaCl_2_ (blue). Germination was monitored at OD_600_ (A, B, C) or by the release of CaDPA in presence of 250 μM Tb^3+^ (D, E, F), respectively. Data points represent the averages from three technical triplicates of biological duplicate experiments and error bars represent the standard error of the mean. (G) Equal numbers of spores were incubated in the indicated conditions (10 mM TA +/- 30 mM co-germinant). Spores were then extracted and separated by SDS-PAGE. SleC activation was analyzed using antisera specific for the SleC protein (the SleC antibody detects the preproSleC, proSleC and activated SleC forms).

In the screen to generate the EMS mutants, the *C*. *difficile* UK1 strain generates more spores than our *C*. *difficile* R20291 isolate, but it is more difficult to genetically manipulate. Thus, to create the *yabG* mutations, we switched to the closely-related *C*. *difficile* R20291 strain. To confirm that the TA-only phenotype is caused by a mutation in *yabG*, we inserted a group II intron into the *yabG* gene of *C*. *difficile* R20291 using TargeTron technology [38, 51, 52]. Germination of the *C*. *difficile yabG*::*ermB* mutant (RS08) spores was compared to wild type using both OD_600_ and CaDPA release assays (Fig 2). As shown in Fig 2A, wild-type *C*. *difficile* R20291 spores required both TA and glycine in order to germinate. Interestingly, though the mutant strain germinates slower, the *yabG*::*ermB* mutant spores germinated in response to TA-only and this germination phenotype was not enhanced by the addition of glycine (in contrast to the phenotype of the EMS-mutant spores) (Fig 2B). We next tested if spores derived from the *yabG*::*ermB* mutant responded to other co-germinants or enhancers of germination. *C*. *difficile* R20291 spores initiated germination in response to TA and L-alanine or TA and CaCl_2_. Significantly, because spores derived from the *yabG*::*ermB* mutant do not respond to L-alanine or to CaCl_2_, this suggested a complete loss of co-germinant recognition. When the mutation was complemented *in trans* by expression of *yabG* from a plasmid (pRS97), the spores again recognized glycine or L-alanine or calcium as a co-germinant (Fig 2C). The TA-only phenotype in the mutant spores was also confirmed by CaDPA release and compared to spores from both wild type and the complemented strain (Fig 2D, 2E and 2F). These results indicate that the *yabG*::*ermB* mutant spores do not respond to co-germinants and germinate in response to TA alone.

In prior work, a *yabG* mutant strain accumulated unprocessed CspBA into spores [43]. To confirm that the generated *yabG* mutant results in the accumulation of CspBA, we purified spores derived from R20291, *yabG*::*ermB* and the complemented strain and separated the extracted protein by SDS-PAGE. The separated protein was then detected using immunoblotting with CspB-specific antisera (S2 Fig). Although the blot showed that little CspBA is still remaining in both the wild type and complemented strains, the *yabG* mutant had mostly the unprocessed, CspBA form. The CspA western blot showed that the *yabG* mutant had only the CspBA form. The mutation in *yabG* did not appear to affect CspC incorporation into the spores although these results are not quantitative.

YabG also was shown to be required for the processing of preproSleC into proSleC [43]. Indeed, whereas the wild-type and complemented strains incorporated into spores the processed, proSleC, form, only preproSleC was incorporated into the *yabG*::*ermB* strain (Fig 2G). When tested for the processing of SleC during germination, the wild-type and the complemented strains required both TA and glycine or TA and L-alanine or TA and calcium to activate SleC (Fig 2G). However, *C*. *difficile yabG*::*ermB* activated SleC in response to TA alone. These results confirm the TA-only phenotype observed in Fig 2B and 2E and suggest that a protein that is not processed in the *yabG* mutant strain is involved in germinant recognition or regulating germinant specificity.

### Quantifying levels of CspB, CspC and SleC in spores from various strains

**Fig 3:**
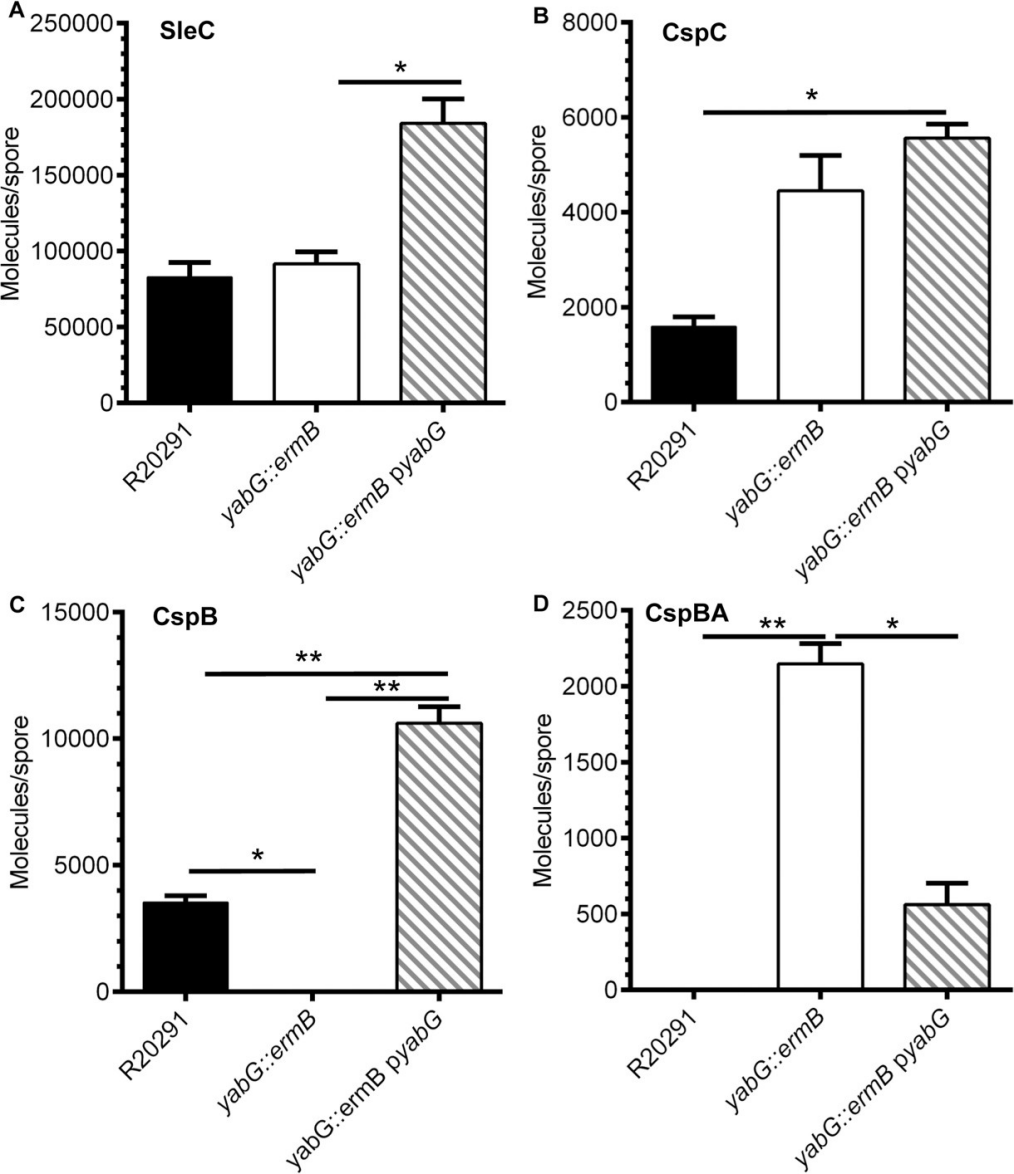
Quantifying the abundance of CspB, CspC and SleC in *C*. *difficile* spores. 2 x 10^9^ spores purified from *C*. *difficile* R20291, the *yabG* mutant and complemented strains were extracted with NuPAGE buffer. Samples were separated by SDS-PAGE, transferred to low fluorescence PVDF membranes and blotted with antisera specific to the indicated proteins. The primary antibody was detected with a fluorescently conjugated secondary antibody and quantified as described in materials and methods. Quantified proteins are expressed as molecules / spore and are represented in a bar graph form. (A) SleC, (B) CspC, (C) CspB and (D) CspBA. The data presented represent the averages from three independent experiments and error bars represent the standard error of the mean. Statistical significance was determined using a 1 way ANOVA with Tukey’s multiple comparisons test (* p < 0.05; ** p < 0.01). *yabG*::*ermB* only incorporates the preproSleC form.

Because YabG is a sporulation-specific protease, it is possible that the deletion of this protease might alter the amount of germination-related proteins (*e*.*g*., CspB, CspC, CspA or SleC) that are incorporated in the spore thereby providing the observed phenotype (*i*.*e*., increasing the abundance of the germinant receptors could lead to an increase in germinant sensitivity and loss of regulation). Using the previously described method to quantify the protein levels in *C*. *difficile* spores [33], we quantified the abundance of CspB (and CspBA), CspC and SleC in *C*. *difficile yabG*::*ermB* and compared them with the abundances in the wild-type and complemented strains (Fig 3). Briefly, equal amounts of spores were boiled in SDS sample buffer to extract the soluble proteins and loaded in the SDS-PAGE gel with recombinantly expressed and purified protein as standards. The separated samples were transferred to low fluorescence-PVDF membrane for quantitative western blot. Membranes were blocked and proteins labeled with antisera specific for the indicated protein. Primary antibodies were detected by incubating with AlexaFluor 555 labeled secondary antibody in the dark. The fluorescently labeled antibodies were detected by scanning on a Typhoon scanner. The fluorescent signal was quantified and compared to the standard curve generated from the recombinant standards included on each gel.

Introducing the *yabG* complementing plasmid resulted in significantly increased abundance of proSleC and CspC into mature spores compared to the *yabG*::*ermB* strain (Fig 3A; only the preproSleC form is incorporated into the *yabG*::*ermB* strain) or the wild-type strain (Fig 3B), respectively. There were no statistical differences in the abundance of SleC or CspC between the wild-type and mutant strains; despite the apparent difference between the WT and *yabG*::*ermB* strains, the CspC abundance did not meet statistical significance. Importantly, there was a statistical difference in the abundance of CspB in all pair-wise comparisons of the wild type and complemented strain (there was no quantifiable CspB protein in spores derived from the *yabG*::*ermB* strain; Fig 3C). Finally, spores derived from the *yabG*::*ermB* strain had significantly more CspBA protein than did the wild-type or the complemented strains (Fig 3D). Because spores derived from the complemented strain had increased abundances of proSleC (Fig 3A), CspC (Fig 3B), and CspB (Fig 3C), but did not produce a TA-only phenotype, this suggests that increased abundance of these proteins is not the reason for the observed TA-only phenotype in the *yabG* mutant strain. Importantly, though, the *yabG* mutant accumulated much more CspBA into spores than did the wild-type or the complemented mutant strains (Fig 3D) and only accumulated the preproSleC form (Fig 2G). Therefore, we hypothesized that the presence of full-length CspBA and / or preproSleC could contribute to the observed TA-only phenotype.

### Deletions in the *cspBA* coding sequence lead to the observed TA-only phenotype

Because CspB and CspC are already known to be involved in regulating *C*. *difficile* spore germination [38, 43–45, 48], we chose to first focus on the potential processing of CspBA by YabG. In prior work by Kevorkian *et al*. [43], the CspBA processing site was hypothesized to occur at or near amino acid 548. To test this hypothesis, we deleted from the *C*. *difficile* CRG2359 (*C*. *difficile* R20291 Δ*pyrE*) chromosome 12 aa between CspB and CspA (*cspBA*_Δ548–560_) using *pyrE*-mediated allelic exchange. After confirmation of the engineered mutation in the CRG2359 genome, the *pyrE* gene was restored, and germination of the resulting strain was compared to *pyrE*-restored CRG2359 strain (*C*. *difficile* RS19; S3A Fig. We found that the CspBA_Δ548–560_ allele did not have any effect on germination (S3B Fig).

**Fig 4:**
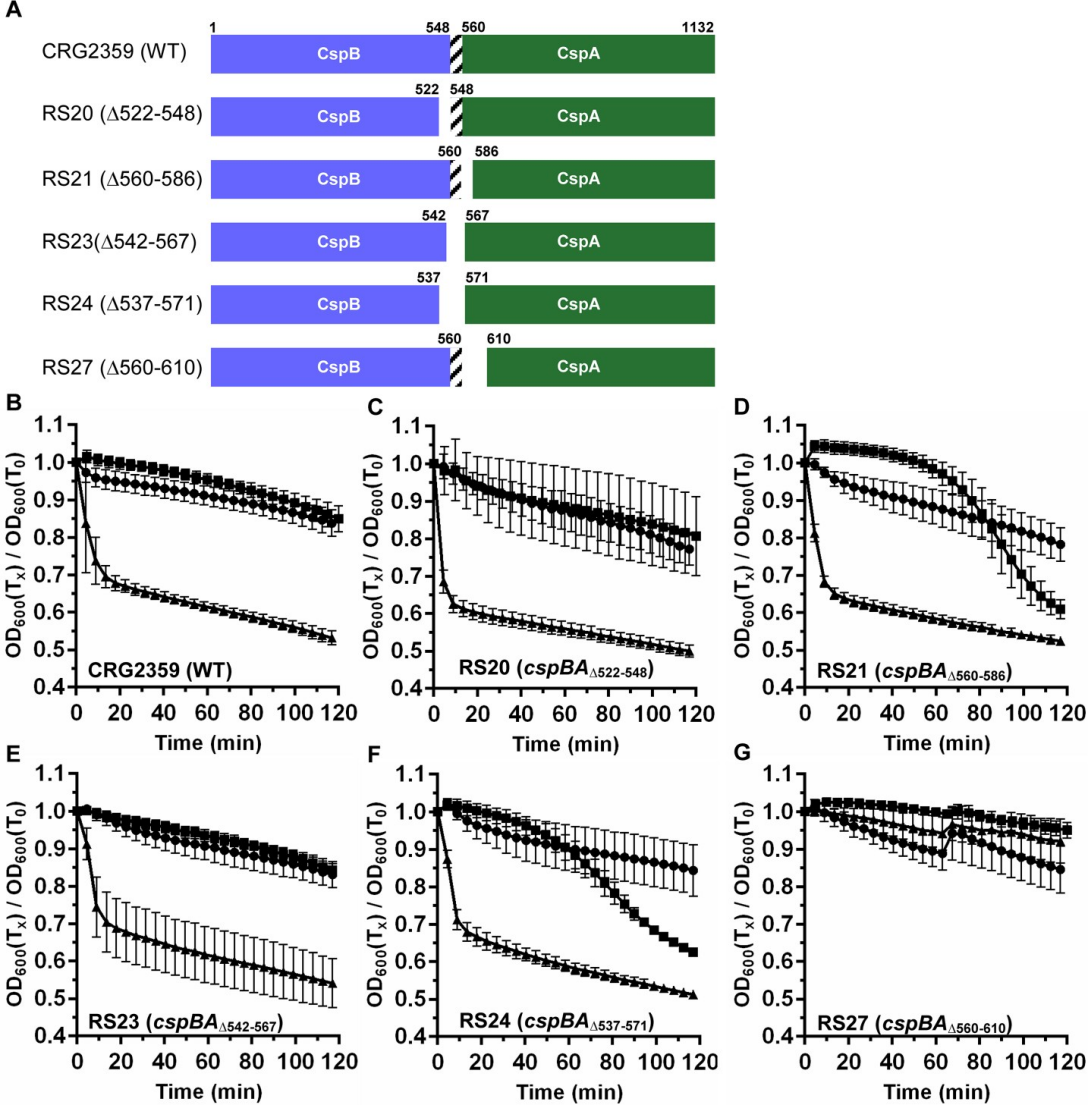
The N-terminus of *C*. *difficile* CspA is important for regulating germination in response to glycine. (A) Graphical representation of the various deletions introduced into *cspBA* compared to the parental, CRG2359; R20291Δ*pyrE* strain. Spore germination of the indicated strain was monitored at OD_600_ in buffer supplemented with (black circle) 30 mM glycine or (black square) 10 mM TA or (black triangle) 10 mM TA and 30 mM glycine. (B) CRG2359, (C) RS20 (*cspBA*_Δ522–548_), (D) RS21 (*cspBA*_Δ560–586_), (E) RS23 (*cspBA*_Δ542–567_), (F) RS24 (*cspBA*_Δ537–571_) and (G) RS27 (*cspBA*_Δ560–610_). Data points represent the averages from three technical triplicates of biological duplicate experiments and error bars represent the standard error of the mean.

Next, to determine if deletions in the coding region in or between *cspB* and *cspA* affect spore germination, we deleted various regions within the *cspBA* gene in the CRG2359 strain (Fig 4A). The results of the germination phenotypes of the various deletions are shown in Fig 4. Deletion of 26 codons from the C-terminus of *cspB* (RS20; CspBA_Δ522–548_) did not affect spore germination (Fig 4C) when compared to the wild-type CRG2359 strain (Fig 4B). These results suggest that the C-terminus of CspB is not involved in generating the TA-only phenotype. Interestingly, deletion of 26 codons at the N-terminus of *cspA* (RS21; CspBA_Δ560–586_) resulted in spores that germinated in response to TA-only, after 60 minutes of incubation in the germination solution (Fig 4D). Though deletion of 25 codons in between the *cspB* and *cspA* coding sequences did not result in a TA-only phenotype (RS23; CspBA_Δ542–567_) (Fig 4E), extending the deleted region by another 9 codons into the surrounding region (RS24; CspBA_Δ537–571_) resulted in spores that germinated in response to TA-only, again after 60 minutes of incubation (Fig 4F). Importantly, the spores derived from the RS21 and RS24 strains still respond to glycine as co-germinant, despite also germinating in response to TA-only. Because the TA-only phenotype appeared to be enhanced as more *cspA* was deleted (RS21 to the RS24 strain), we predicted that a larger deletion might result in a phenotype similar to that observed in the *yabG* mutant (which does not recognize co-germinants). We found that when 50 codons were deleted from the N-terminus of CspA (RS27) the spores no longer germinated (Fig 4G). These results were confirmed by analyzing the release of CaDPA from the germinating spores (S4 Fig).

During construction of the deletion strains, we encountered significant difficulties in restoring the *pyrE* allele to wild type. To circumvent this obstacle, and to understand if *C*. *difficile* spore germination is affected by the *pyrE* deletion, we restored *pyrE* in the RS21 strain (CspBA_Δ560–586_, *pyrE*^+^; RS26) and compared with the RS19 strain (CRG2359 with restored *pyrE*). As shown in S5A Fig, *C*. *difficile* RS19 required both TA and glycine to germinate but the RS26 strain germinated in response to TA-only (S5B Fig). These results were confirmed by analyzing the release of CaDPA from the spore (S5C and S5D Fig). Finally, we analyzed the activation of proSleC to SleC in response to TA alone. Only the RS26 strain cleaved proSleC in response to TA alone (S5E Fig). These observations are identical to the observations made for the parental RS21 strain (Fig 4 and S4 Fig) and indicate that the *pyrE* allele does not influence *C*. *difficile* spore germination in the context of these studies.

**Fig 5:**
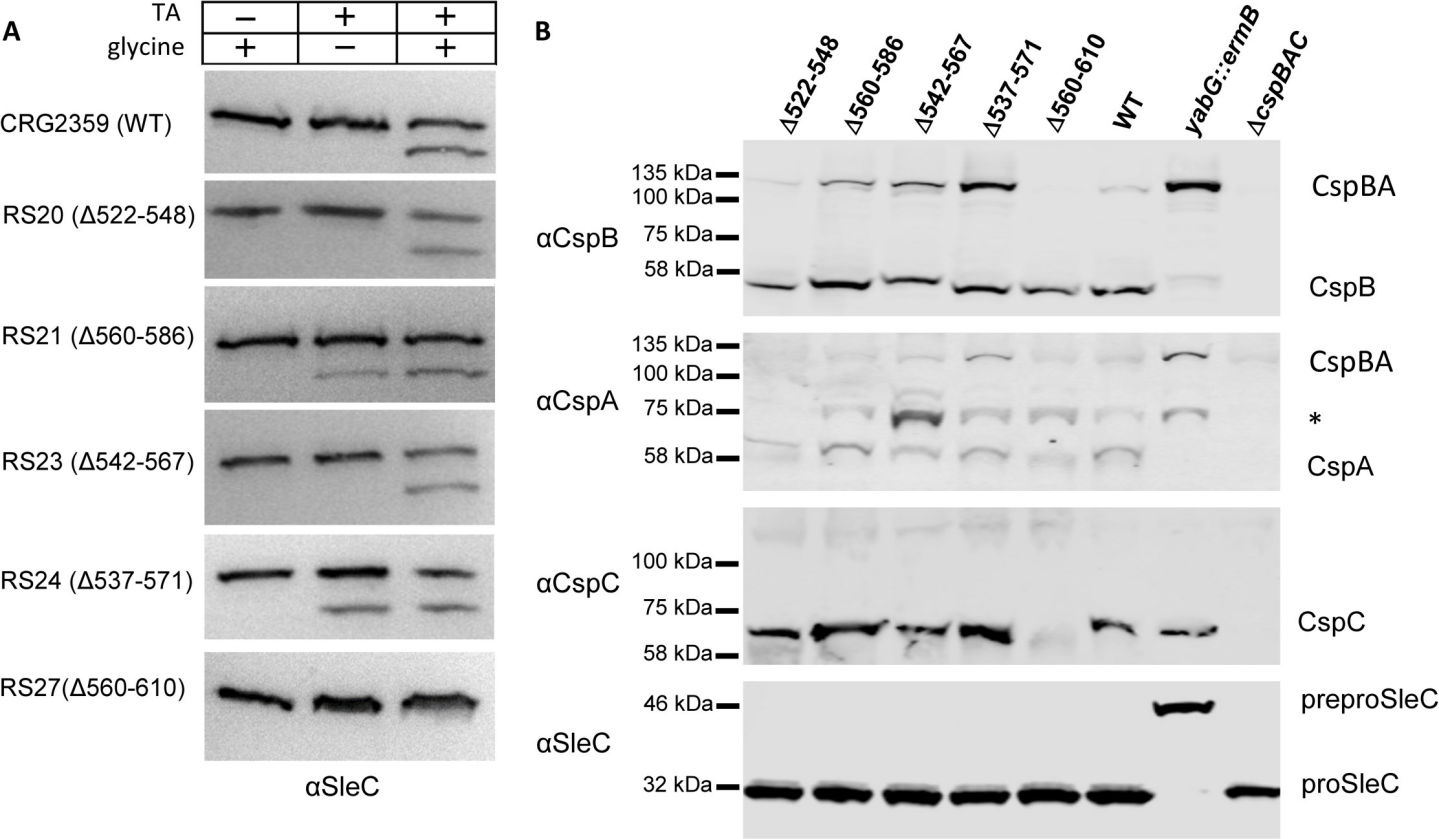
Comparing the effects of mutations in *C*. *difficile cspBA* on the incorporation and processing of CspB, CspA, CspC and proSleC. Spores derived from the indicated strains were extracted and separated as described in Fig 2. (A) SleC activation was measured in buffer supplemented with 30 mM glycine or 10 mM TA or both 10 mM TA and 30 mM glycine for 2 hours at 37°C and (B) CspB, CspA, and CspC were detected as described in Fig 2 (* alternative processing of CspA).

To confirm our observations that the strains germinated in response to TA alone, we analyzed by western blot the activation of SleC (Fig 5A). SleC activation in response to 10 mM TA only occurred in CspA_Δ560–586_ and CspA_Δ537–571_ while CspA_Δ560–610_ did not germinate in response to TA and glycine. The CspB western blot showed that CspBA was processed to CspB and CspA in all of the mutants, compared to wild type (Fig 5B). The CspC western blot did not reveal any differences between the wild type and mutant strains, except for the RS27 (CspA_Δ560–610_) strain where no CspC was detected.

### Deletion of a hypothesized YabG cleavage site in CspA and preproSleC results in the TA-only phenotype

**Fig 6:**
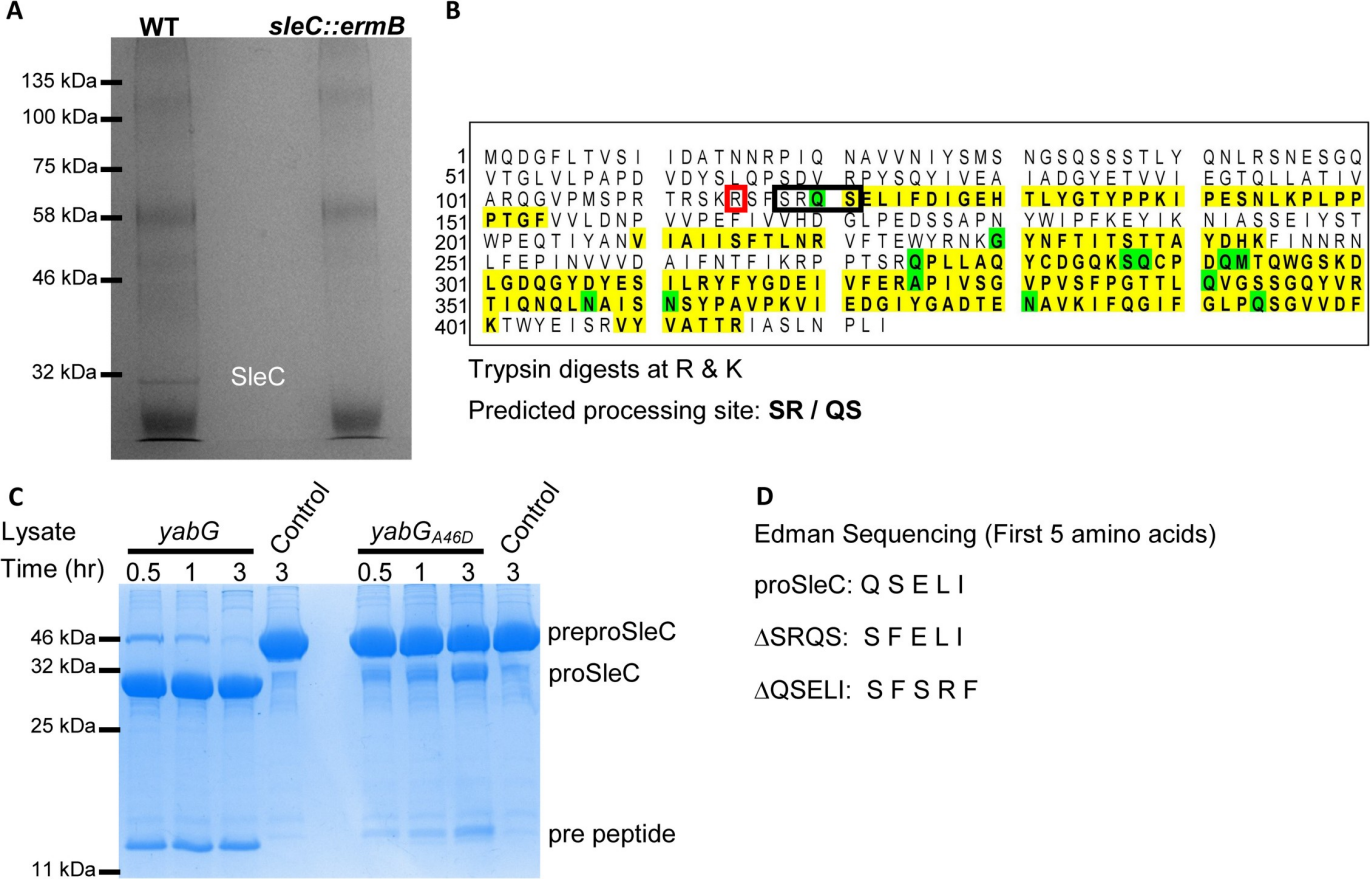
Identifying and characterizing the YabG processing site in preproSleC. (A) Spores derived from *C*. *difficile* R20291 (WT) and *C*. *difficile* RS10 (*sleC*::*ermB*) were disrupted by bead-beating and then proSleC was immunoprecipitated using SleC-specific antisera. Samples were separated by SDS-PAGE gel and stained with Coomassie blue. The band corresponding to proSleC was excised from the gel and analyzed by peptide mass finger printing. (B) The sequence of preproSleC is listed. Yellow highlighted regions correspond to fragments that were detected by mass spectrometry and amino acids highlighted in green indicate amino acids that are modified in the MS analysis (*i*.*e*., oxidized, deaminated, acetylated or ammonia loss). (C) Recombinantly expressed and purified preproSleC was incubated for the indicated times in *E*. *coli* lysate that expressed either wildtype *yabG* or *yabG*_*A46D*_ (Table 2). A control sample of recombinant protein incubated in buffer was included. Samples were boiled in SDS-sample buffer and separated by SDS-PAGE before staining with Coomassie Blue. (D) Recombinantly expressed and purified preproSleC, preproSleC_ΔSRQS_ or preproSleC_ΔQSELI_ were incubated with wildtype YabG as in (C). The first five amino acids at the N-terminus were determined by Edman sequencing. R_115_ is highlighted in red.

Previous work has shown that CspA has a role in either incorporating CspC or stabilizing CspC in mature spores [43]. The data presented above suggests that the N-terminus of CspA is important for regulating spore germination. We had hypothesized that this region is processed by YabG but all the generated *cspA* alleles generated a protein that was processed into the CspB and CspA forms (Fig 5). One way to identify the YabG processing site in CspA is by pulldown of CspA from the spore extract and sequencing the CspA protein using mass spectrometry or Edman sequencing. Unfortunately, the CspA antibody was unable to immunoprecipitate CspA from the spores due to the quality of the antibody. However, we predicted that the YabG processing site in CspBA might be conserved in preproSleC. Instead of immunoprecipitating CspA, we immunoprecipitated proSleC from spore extracts derived from wild-type spores and *sleC* mutant spores (as a negative control) (Fig 6A). Using the sample from the proSleC pull down, we identified fragments by mass spectrometry of trypsin-digested proSleC (Fig 6B). In this experiment, the most N-terminal fragment identified began with glutamine_120_ followed by serine_121_ (an SR**Q**S sequence), suggesting that YabG cleaved after R_119_. However, because the protein was digested with trypsin, which cleaves after arginine and lysine residues, this N-terminal glutamine could be the result of trypsin cleavage and not YabG processing of preproSleC during spore development. Therefore, we recombinantly expressed and purified preproSleC from *E*. *coli*. This recombinant protein was incubated with an *E*. *coli* lysate that express *yabG* from an IPTG-inducible promoter for 30 minutes, 1 hour, 3 hours or in buffer alone for 3 hours (Fig 6C). This lysate efficiently processed preproSleC to a form consistent with the removal of the pre sequence and this did not occur in buffer alone. Interestingly, when preproSleC is incubated in *E*. *coli* lysate that expressed one of the *yabG* alleles identified in the EMS-mutagenesis (*yabG*_*A46D*_), preproSleC was not processed efficiently. This indicates that the EMS-generated mutant expresses a less active YabG protein and that the *in vitro* processing of the preproSleC is due to YabG and not some other *E*. *coli* protease. Using the protein processed by wildtype YabG, we submitted the processed protein for Edman sequencing (Fig 6D).

Edman sequencing of the YabG-processed preproSleC protein revealed that the first five amino acids were QSELI (Fig 6D). This sequence matched the mass spectrometry data and confirms that YabG processes preproSleC after R_119_. These results are similar to what is observed in *C*. *perfringens* preproSleC cleavage (*C*. *perfringens* preproSleC is processed after R_114_) [53]. Next, we tested the impact of deletion of the SRQS sequence or the QSELI sequence from preproSleC. These protein alleles were recombinantly expressed and purified and then incubated with *E*. *coli* lysate that expressed *yabG*. The resulting protein was submitted for Edman sequencing. Surprisingly, we found that deletion of the SRQS sequence resulted in YabG cleaving after R_115_ and the N-terminus of proSleC being SFELI and deletion of QSELI also resulted in YabG cleaving after R_115_ and the N-terminus being SFSRF. These results indicate that deletion of the YabG cleavage site, or surrounding region, results in YabG processing the protein near the same site. These results suggest that YabG may recognize the structure of its substrates and not a specific amino acid sequence.

**Fig 7:**
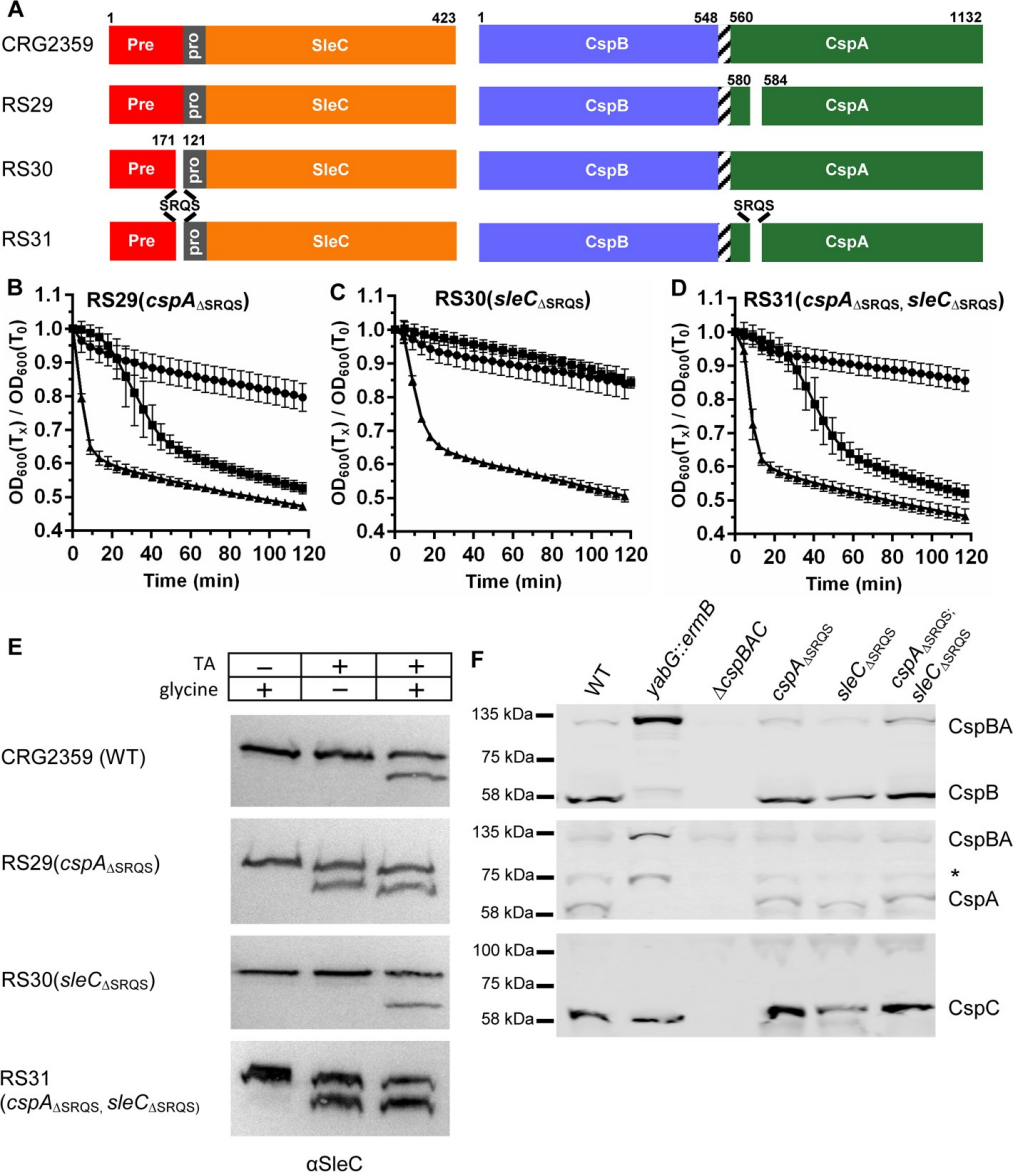
Deletion of the hypothesized YabG cleavage site in CspA results in a TA-only phenotype. (A) Graphical representation of the various deletions introduced into *cspBA* or *sleC* compared to the parental, CRG2359; R20291Δ*pyrE* strain. Spore germination of the indicated strain was monitored at OD_600_ in buffer supplemented with (black circle) 30 mM glycine or (black square) 10 mM TA or (black triangle) 10 mM TA and 30 mM glycine. (B) RS29 (*cspA*_ΔSRQS_), (C) RS30 (*sleC*_ΔSRQS_), (D) RS31 (*cspA*_ΔSRQS_, *sleC*_ΔSRQS_). Data points represent the averages from three technical triplicates of biological duplicate experiments and error bars represent the standard error of the mean. (E) Spores derived from the indicated strains were extracted and separated as described in Fig 2 and SleC activation was measured in buffer supplemented with 30 mM glycine or 10 mM TA or both 10 mM TA and 30 mM glycine for 2 hours at 37°C. (F) CspB, CspA, and CspC were detected as described in Fig 2 (* alternative processing of CspA).

When we compared this identified SRQS sequence to the protein sequence in CspA, we found that within the N-terminus of CspA there was a SRQS amino acid sequence that was encompassed within the deletions found in the RS21 strain (CspA_Δ560–586_; a strain that generated a TA-only phenotype). We deleted the nucleic acid sequence that encodes this SRQS motif in *sleC*, *cspBA*, or in both genes (Fig 7A). Surprisingly, the deletion of the SRQS site in CspA resulted in spores that germinated in response to TA-only within 40 minutes after germinant addition (Fig 7B). However, deletion of the SRQS motif within preproSleC did not affect spore germination (Fig 7C). When these two deletions were combined in the same strain, the spores had a TA-only phenotype similar to that of spores with a deletion in CspA alone (Fig 7D and 7A). Next, we confirmed these phenotypes by analyzing the release of CaDPA from the spore (S6 Fig). Again, only when the *cspA*_ΔSRQS_ allele was incorporated into spores did the resulting strains release CaDPA in response to TA-only (S6A and S6C Fig); *sleC*_ΔSRQS_ spores required both TA and glycine to release CaDPA (S6B Fig).

We next analyzed the activation of SleC for these deletions. proSleC was activated in response to TA-only in the RS29 (*cspA*_ΔSRQS_) and the RS31 (*cspA*_ΔSRQS_ + *sleC*_ΔSRQS_) strains but not in the RS30 (*sleC*_ΔSRQS_) strain (Fig 7E). There was no difference in the CspB or CspC incorporation in these SRQS deletion mutants compared to wild type (CRS2359; Fig 7F). However, as predicted by the processing of preproSleC by YabG (Fig 6), deletion of the SRQS sequence in CspA resulted in processing of CspBA to CspB and CspA (Fig 7F). Our results indicate that due to mis-processing of CspBA, or alterations within the *cspA* sequence, spores lose the requirement for co-germinants during spore germination.

## Discussion

Previous studies on *C*. *difficile* spore germination have hypothesized the presence of an amino acid co-germinant receptor [13, 31, 32, 50]. We used EMS mutagenesis to screen for *C*. *difficile* mutants whose spores recognize betaine as a germinant. Excitingly, we isolated mutants that did not require an amino acid as a co-germinant, and these EMS-mutants germinated in response to TA-only. However, these mutants still recognized co-germinants; the rate of germination increased upon the addition of co-germinant to the germination solution. We traced the SNP to the *yabG* locus and confirmed that the TA-only phenotype observed in the EMS-generated mutants was due to the *yabG* allele. In contrast to the EMS-generated mutants, the *yabG* mutant spores did not recognize glycine as a co-germinant, and germinated in response to TA-only (Fig 2B and 2C). Oddly, we noticed that spores derived from the complemented strain released some CaDPA in response to TA alone, but this was not observed when germination was measured at OD_600_ (Fig 2F vs. 2E). We further analyzed this observation and found that the supposed CaDPA release observed in the complemented strain in the presence of TA alone, was due the presence of Tb^3+^ in the assay (Tb^3+^ is absent from the germination solution in the OD_600_ assay). We have submitted a separate manuscript to report this in detail.

To understand if spores derived from the *yabG* mutant recognized other amino acids as co-germinants, we tested L-alanine and calcium. L-alanine is the second-best amino acid co-germinant during *C*. *difficile* spore germination with an EC_50_ value of 5 mM [32, 50]. Because *C*. *difficile* RS08 (*yabG*::*ermB*) spores did not respond to glycine or L-alanine or CaCl_2_ (Fig 2), these results suggest that the protein(s) YabG processes is / are responsible for recognition of the various co-germinant(s).

YabG has been mostly studied in *B*. *subtilis*. In *B*. *subtilis* YabG is a sporulation-specific protease, and a *yabG* mutation causes alterations in the coat proteins of *B*. *subtilis* spores. The orthologues of most *B*. *subtilis* YabG target proteins are absent in *C*. *difficile* (*e*.*g*., CotT, YeeK, YxeE, CotF, YrbA) [54–56]. In a previous report, the processing of CspBA and preproSleC during *C*. *difficile* sporulation was shown to be YabG-dependent [43]. However, germination was not significantly altered in the mutant spores compared to wild type spores (germination efficiency decreased from 1 to 0.8 in mutants, when analyzing CFU counts) [43]. Importantly, though, germination efficiency was only tested on BHIS-TA agar medium and not in the presence of TA alone [43].

In prior work by Kevorkian *et al*. [43], the authors suggested that the CspBA processing site is near-amino acid 548 and is encoded by a linker DNA sequence between *cspB* and *cspA*. To test this, we deleted the codons encoding amino acids 542–567 regions in *cspBA* (RS23). When the germination phenotype of the RS23 strain was compared with CRG2359, we did not observe a TA-only phenotype (Fig 4B and 4E) and, importantly, CspBA was still efficiently processed (Fig 5). These results suggest that the predicted 548–560 region as the YabG-dependent processing site is not accurate, or that when the YabG processing site is removed, YabG cleaves the CspBA protein at an alternate site. In support of this hypothesis, we found that deletion of the YabG processing site in preproSleC resulted in YabG cleaving positionally within the protein. This is likely to be the case for CspBA processing as well. Further work is needed to understand how a protein that is found in all spore-forming bacteria, YabG, recognizes its substrates, which are diverse.

When the identified SRQS sequence was deleted from CspA (*cspBA*_Δ580–584_), we observed a TA-only phenotype in the spores suggesting that the SRQS region might be important for CspA activity. Both RS21 (*cspBA*_Δ560–586_) and RS24 (*cspBA*_Δ537–571_) strains generated spores with TA-only phenotypes (Fig 4) similar to the SRQS deletion in CspA (Fig 7). However, only the RS21 strain has the deletion of SRQS region, while the SRQS motif is still present in the RS24 strain.

In a recent review on *C*. *difficile* spore germination [13], the authors build upon a hypothesized model for spore germination [10] and propose a new, “lock and key” model for *C*. *difficile* spore germination. In the “lock and key” model, CspA and CspC are localized in the coat layer, where TA can bind to CspC. Activation of CspC leads to the transport of glycine and Ca^2+^ through the outer membrane, by an unknown protein, to the cortex where CspB is held inactive in a complex with GerS and proSleC. In the absence of calcium, glycine is transported to the inner membrane where it activates the release of Ca^2+^ from the core through another unknown process. The released Ca^2+^ traffics to CspB to activate its protease activity. In the presence of calcium and glycine, both are transported in and Ca^2+^ activates CspB. When glycine and calcium bind to CspB, CspB can activate proSleC to degrade the cortex [13]. In the first, favored, model, the germinosome complex composed of CspC, CspA, CspB, and proSleC are anchored to the outer spore membrane by GerS (a lipoprotein that is required for spore germination [57]—see below) [10, 13]. Upon binding of a germinant (TA with glycine or Ca^2+^), CspC and CspA are released from this germinosome complex and CspB becomes free to activate proSleC to degrade the cortex. Subsequently, CaDPA is released from the spore core by a mechanosensing mechanism [26, 46].

Our results support the first model [10] whereby CspB, CspA, and CspC are in a germinosome complex–similar to the germinosome complex found in *B*. *subtilis* [58]. Recent studies on GerS have shown that GerS likely does not form a complex with other germination proteins [57, 59]. Rather, it is required to generate cortex-specific modifications in the spore and thus, important for germination of the spore because the SleC cortex lytic enzyme depends on cortex-specific modifications to degrade the cortex layer efficiently [59]. Based upon prior work from our lab, proSleC is unlikely to be part of this complex because SleC is three to four times more abundant than CspB or CspC, depending upon the strain analyzed [33].

The findings by Kochan and colleagues that calcium synergizes with other co-germinants during *C*. *difficile* spore germination are interesting [37]. It is possible, however, that what the authors are observing is that the population of spores is germinating in response to TA and amino acids or in response to TA and calcium and that individual spores within that population are responding to either glycine or to calcium as co-germinants. Because spores derived from the *yabG*::*ermB* strain lose all requirements for co-germinant (either amino acids or calcium), we hypothesize that CspA is recognizing all co-germinants (amino acids or divalent cations) for *C*. *difficile* spores. Though the evidence for this hypothesis is entirely genetic, this hypothesis provides a model that can be tested biochemically (once reagents are built for such experiments).

**Fig 8:**
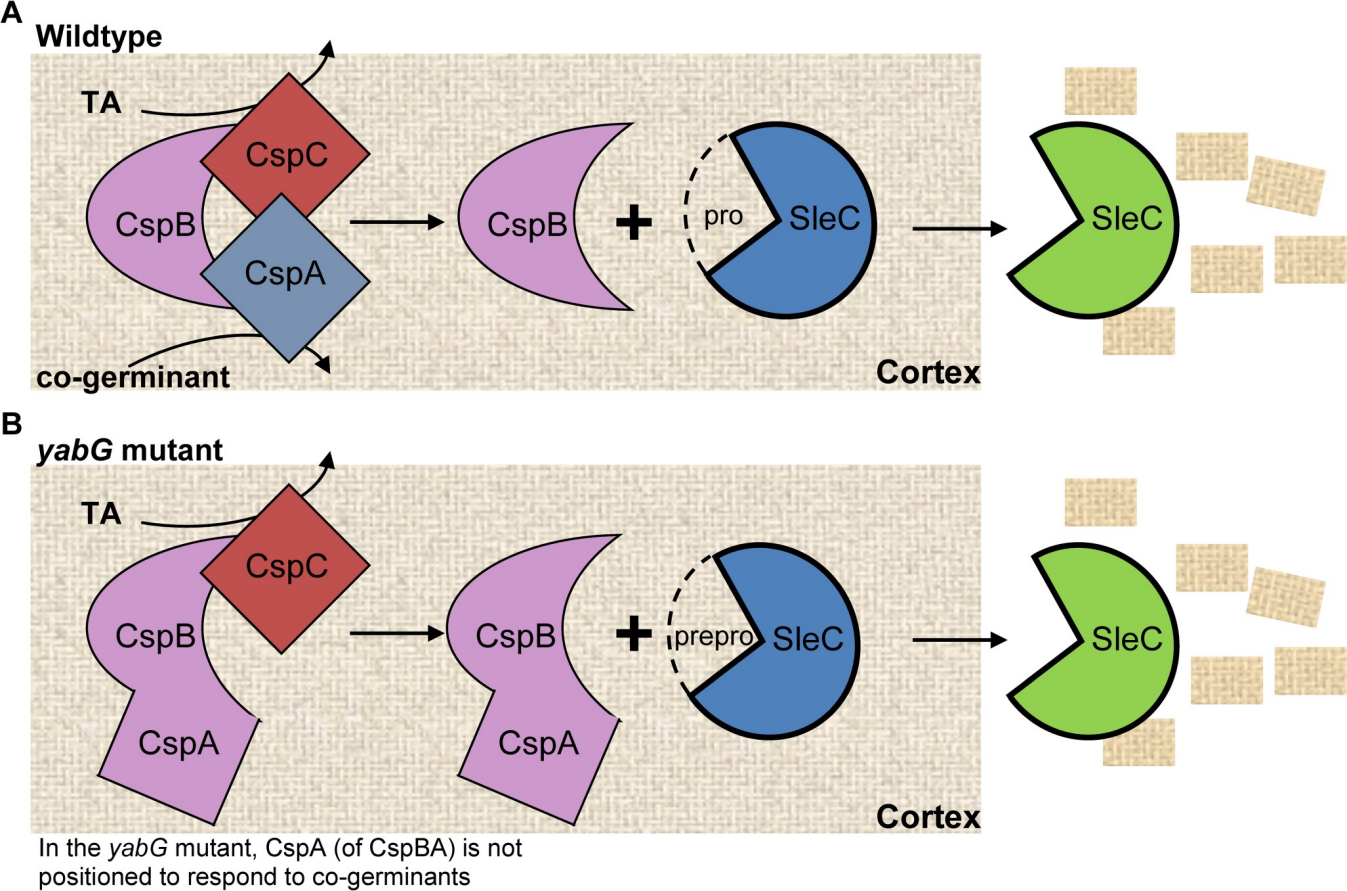
Model for *C*. *difficile* spore germination. (A) In our working model for wildtype *C*. *difficile* spore germination, CspB protease activity is inhibited by two pseudoproteases, CspC (the bile acid germinant receptor; [38]) and CspA (implicated as the co-germinant receptor; this study). Interaction of CspC with cholic acid derivatives [31] and the interaction of CspA with co-germinants [32, 68] results in these two proteins disassociating from CspB. Subsequently, CspB cleaves the inhibitory pro-domain from proSleC thereby activating SleC’s cortex degrading activity. (B) In the *C*. *difficile yabG*::*ermB* mutant, CspBA is not processed into the two proteins important for *C*. *difficile* spore germination. In this scenario, the CspA portion is not positioned correctly to inhibit CspB activity until the co-germinant signal is received. This results in CspB activity being inhibited only by CspC. In response to TA, CspC dissociates from CspBA and CspB (of CspBA) processes preproSleC to the active, SleC, form.

The processing of CspBA depends on the YabG protease and *yabG*-mutant spores incorporate mostly the full length CspBA protein. These spores do not recognize amino acids as co-germinants and germinate in response to TA-only (Fig 2B). We hypothesize that CspC (functioning as the bile acid germinant receptor) and CspA (functioning as the amino acid co-germinant receptor) inhibit CspB activity within dormant spores (Fig 8A). These two pseudoproteases would regulate the activity of CspB so that it does not prematurely activate proSleC, potentially similar to how other pseudoproteases / pseudokinases regulate activity of their cognate proteins [60–64]. Should proSleC become activated prematurely, the *C*. *difficile* spore could germinate in an environment that may not support growth (*e*.*g*., absence of glycine, or other amino acids, or absence of divalent cations).

Pseudoenzymes have gained attention, recently, for regulating biological processes. For example, pseudokinases have been studied in both prokaryotes [*e*.*g*., *Caulobacter crescentus* DivL [65] or *Streptococcus pyogenes* RocA [66]] and eukaryotes [60–63] where they often regulate their cognate protease / kinase. Pseudoproteases, however, have mostly been studied in eukaryotes [64]. Prior work by Hershey and colleagues [67] has revealed that *Magnetospirillum magneticum* uses a pseudoprotease, MamO, to bind metals for magnetosome development. Combined with this study, our work provides growing evidence that pseudoproteases also regulate biological processes in prokaryotes.

Although some aberrantly processed CspB is detectable in the *yabG* mutant spore, the spore packages mostly full-length CspBA, where CspA is tethered to CspB. We hypothesize that in this scenario, CspC alone prevents CspB from cleaving proSleC into its active form, and TA might dislodge CspC from CspB (this is consistent with our prior publication that indicates that CspC may have an inhibitory activity during spore germination [33]) (Fig 8B). Moreover, our data suggest that the N-terminus of CspA might be important for formation of this hypothesized complex with CspC and / or CspB. When portions of the CspA N-terminus are deleted, the binding of CspA to the complex might become unstable causing CspA to randomly disassociate from the complex. This would result in TA alone stimulating germination by disassociating CspC from CspB (Fig 8B). Once CspB is free from the complex, it could then activate many proSleC proteins to maximize the germination process. Further work is needed to test the biochemical implications of this hypothesis.

## Supporting information

S1 Fig Biological replicates of the germination plots in Fig 1. The germination phenotype of wild type *C*. *difficile* UK1 and mutant spores were screened by measuring CaDPA release in presence of (black circle) 30 mM glycine, (black square) 10 mM TA or (black triangle) 10 mM TA and 30 mM glycine.
(TIF)

S2 Fig CspB, CspA and CspC incorporation into *C. difficile* R20291, *yabG::ermB*, *yabG::ermB* p*yabG* and Δ*cspBAC* strains. Purified *C*. *difficile* spores from the indicated strains were extracted as described in the materials and methods. The resulting protein was separated by SDS-PAGE in immunoblotted with antisera raised against the indicated protein. A cross reactive protein (*) is present in the CspA immunoblot that nearly overlaps with the CspBA form of the protein.
(TIF)

S3 Fig CaDPA release for *C. difficile* CRG2359 *pyrE^+^* (RS19) and *cspBA*_Δ548–560_ (RS18). CaDPA release from spores purified from *C*. *difficile* CRG2359 with restored *pyrE* (A) and RS18 (*cspBA*_Δ548–560_; restored *pyrE*) (B) strains was analyzed by suspending the spores in buffer supplemented with 250 μM Tb^3+^ and (black circle) 30 mM glycine or (black square) 10 mM TA or (black triangle) 10 mM TA and 30 mM glycine.
(TIF)

S4 Fig Monitoring CaDPA release from spores with defined deletions in *cspBA*. CaDPA release during spore germination of the indicated strain was monitored by suspending spores in buffer supplemented with Tb^3+^ and (black circle) 30 mM glycine or (black square) 10 mM TA or (black triangle) 10 mM TA and 30 mM glycine. (A) CRG2359, (B) RS20 (*cspBA*_Δ522–548_), (C) RS21 (*cspBA*_Δ560–586_), (D) RS23 (*cspBA*_Δ542–567_), (E) RS24 (*cspBA*_Δ537–571_) and (F) RS27 (*cspBA*_Δ560–610_). Data points represent the averages from three technical triplicates of biological duplicate experiments and error bars represent the standard error of the mean.
(TIF)

S5 Fig Comparing germination and western blots for spores purified from wild type and RS26. (A,B) Spores purified from *C*. *difficile* RS19 (CRG2359 with restored *pyrE*) and RS26 (*cspBA*_Δ560–586_; restored *pyrE*) strains were suspended in buffer supplemented with (black circle) 30 mM glycine or (black square) 10 mM TA or (black triangle) 10 mM TA and 30 mM glycine and OD_600_ was monitored over time. (C, D) CaDPA release from the indicated strains was determined as in A & B with buffer also supplemented with 250 μM Tb^3+^. Data points represent the averages from three technical triplicates of biological duplicate experiments and error bars represent the standard error of the mean. (E) The activation of SleC from spores purified from R20291, RS19 and RS26 in presence of buffered 30 mM glycine or 10 mM TA or both 10 mM TA and 30 mM glycine was determined after incubation for 2 hours at 37°C. (F) Equal numbers of spores derived from R20291, RS19 or RS26 were extracted, separated by SDS-PAGE and transferred to PVDF membranes for immunoblotting of CspB, CspA and CspC.
(TIF)

S6 Fig CaDPA release for the SRQS deletion mutants. CaDPA release during spore germination of the indicated strain was monitored by suspending spores in buffer supplemented with Tb^3+^ and (black circle) 30 mM glycine or (black square) 10 mM TA or (black triangle) 10 mM TA and 30 mM glycine. (A) RS29 (*cspA*_ΔSRQS_), (B) RS30 (*sleC*_ΔSRQS_), (C) RS31 (*cspA*_ΔSRQS_; *sleC*_ΔSRQS_). Data points represent the averages from three technical triplicates of biological duplicate experiments and error bars represent the standard error of the mean.
(TIF)

S1 Table Oligonucleotides used in this study. The oligonucleotides used in this study are listed.
(DOCX)
