# Supplementary figures and images for: The requirement for co-germinants during Clostridium difficile spore germination is influenced by mutations in yabG and cspA
Source: PLoS Pathog. 2019 Apr 3;15(4):e1007681. doi: 10.1371/journal.ppat.1007681 (PMC6464247; doi:10.1371/journal.ppat.1007681)

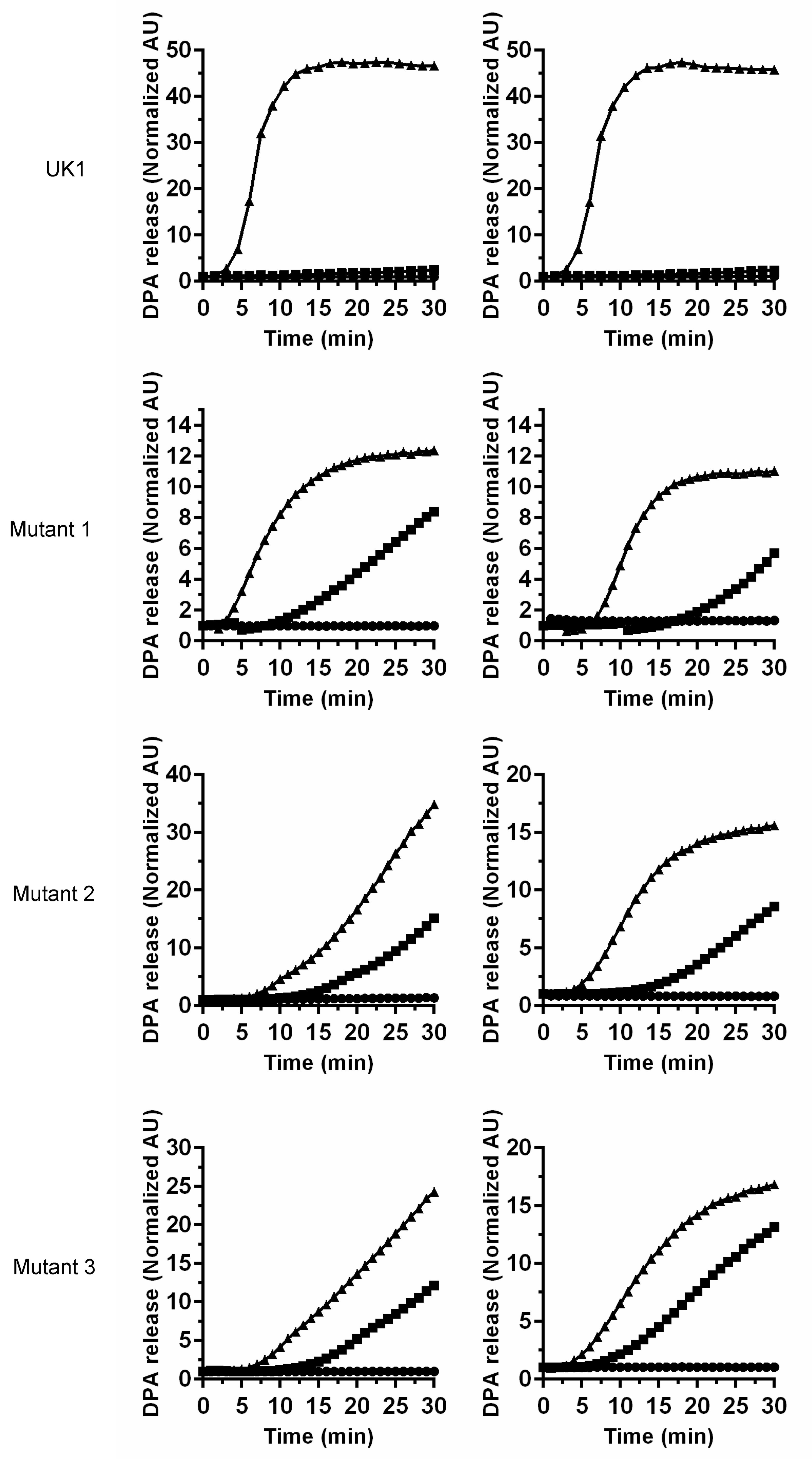

Supplement: S1 Fig — The germination phenotype of wild type C. difficile UK1 and mutant spores were screened by measuring CaDPA release in presence of (black circle) 30 mM glycine, (black square) 10 mM TA or (black triangle) 10 mM TA and 30 mM glycine. (TIF) [file ppat.1007681.s001.tif]

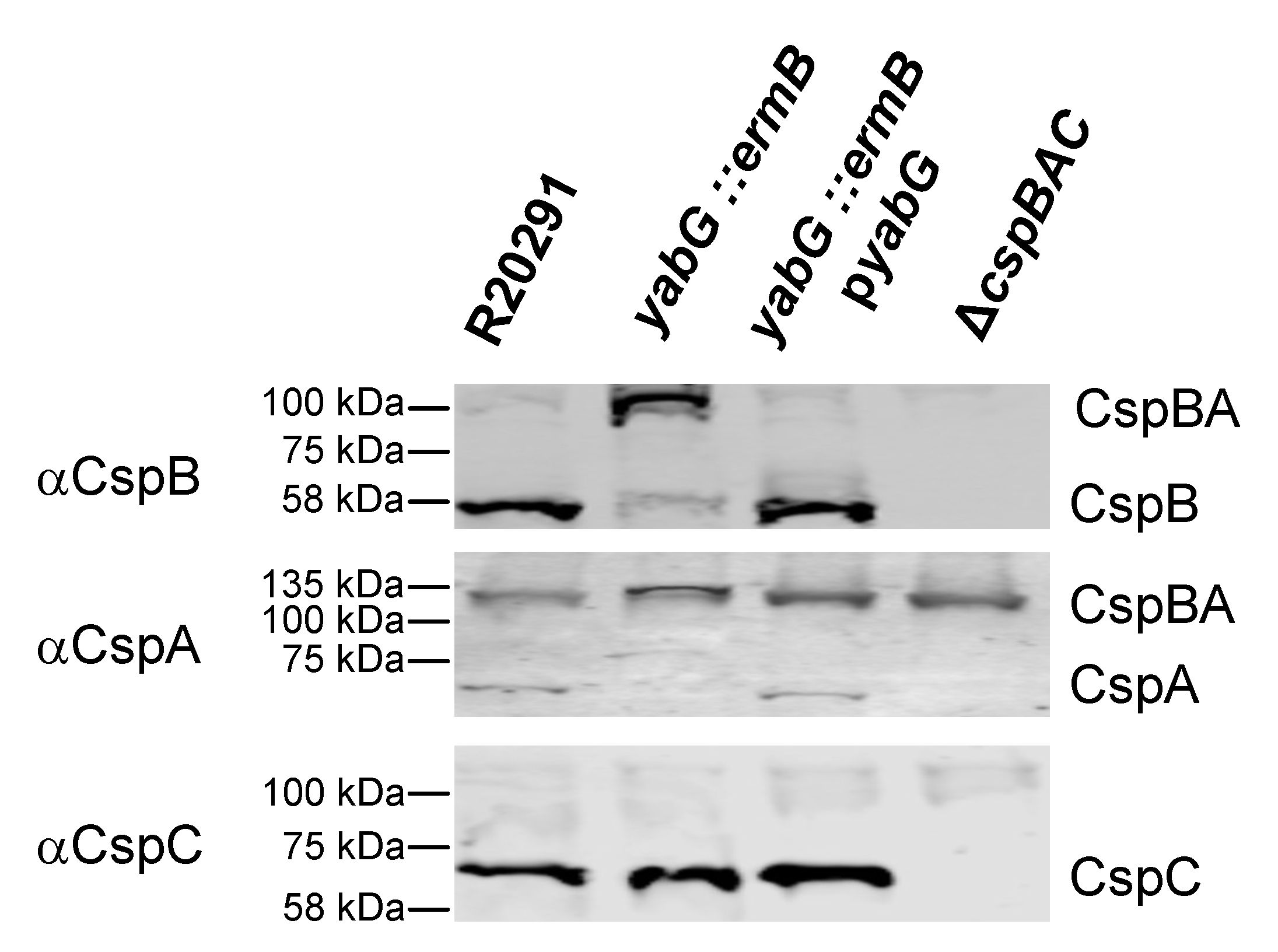

Supplement: S2 Fig — Purified C. difficile spores from the indicated strains were extracted as described in the materials and methods. The resulting protein was separated by SDS-PAGE in immunoblotted with antisera raised against the indicated protein. A cross reactive protein (*) is present in the CspA immunoblot that nearly overlaps with the CspBA form of the protein. (TIF) [file ppat.1007681.s002.tif]

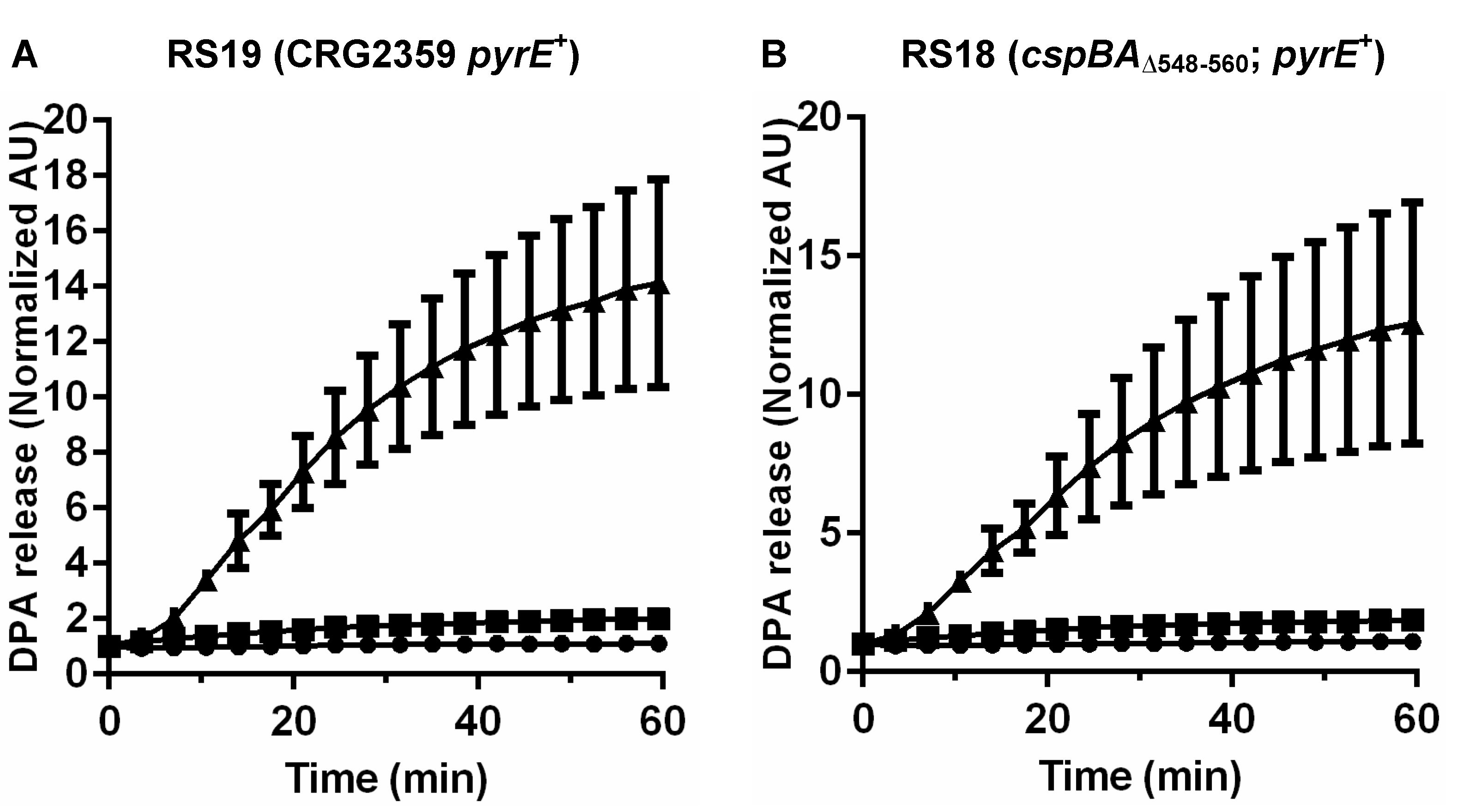

Supplement: S3 Fig — CaDPA release from spores purified from C. difficile CRG2359 with restored pyrE (A) and RS18 (cspBAΔ548–560; restored pyrE) (B) strains was analyzed by suspending the spores in buffer supplemented with 250 μM Tb3+ and (black circle) 30 mM glycine or (black square) 10 mM TA or (black triangle) 10 mM TA and 30 mM glycine. (TIF) [file ppat.1007681.s003.tif]

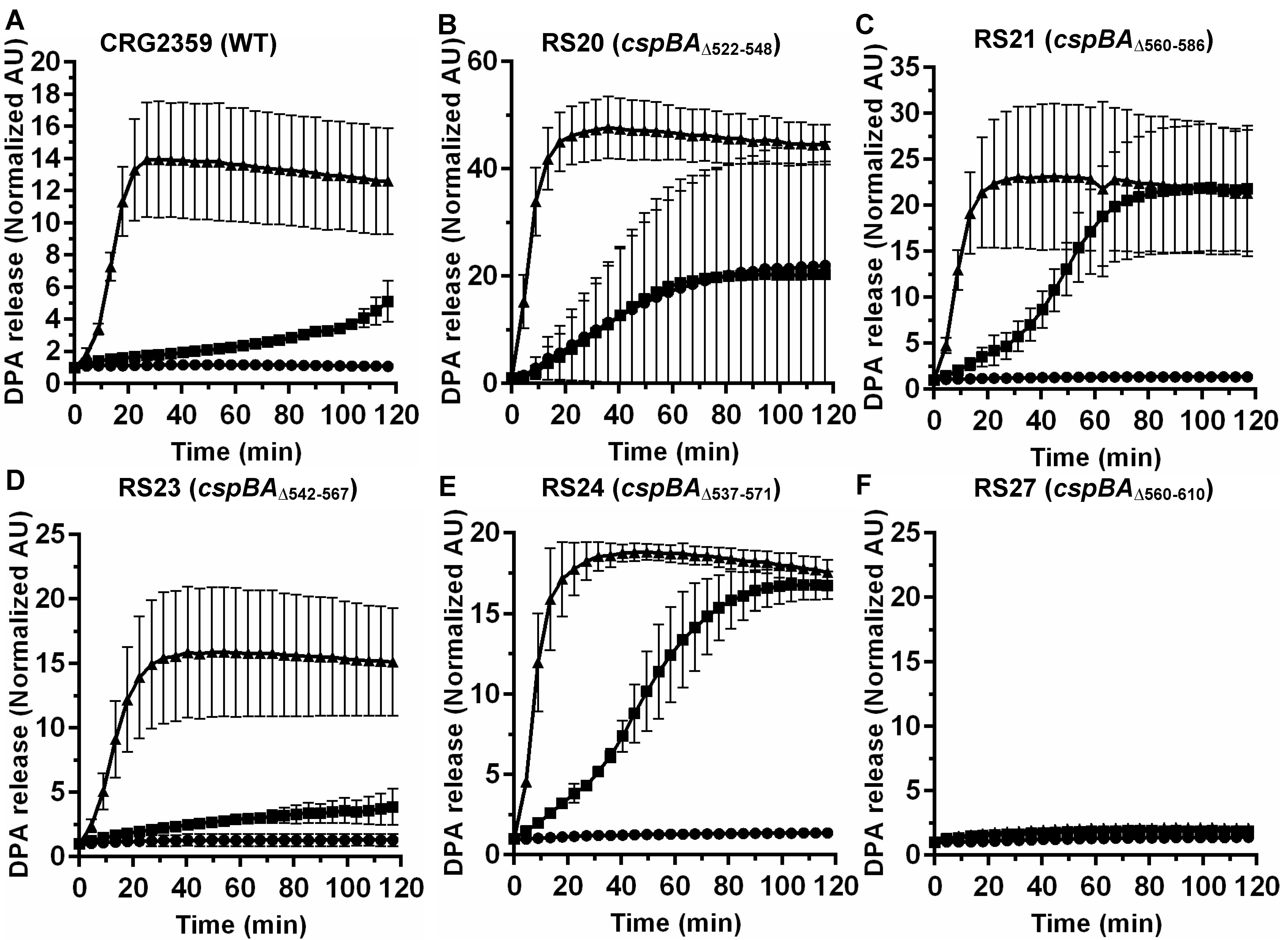

Supplement: S4 Fig — CaDPA release during spore germination of the indicated strain was monitored by suspending spores in buffer supplemented with Tb3+ and (black circle) 30 mM glycine or (black square) 10 mM TA or (black triangle) 10 mM TA and 30 mM glycine. (A) CRG2359, (B) RS20 (cspBAΔ522–548), (C) RS21 (cspBAΔ560–586), (D) RS23 (cspBAΔ542–567), (E) RS24 (cspBAΔ537–571) and (F) RS27 (cspBAΔ560–610). Data points represent the averages from three technical triplicates of biological duplicate experiments and error bars represent the standard error of the mean. (TIF) [file ppat.1007681.s004.tif]

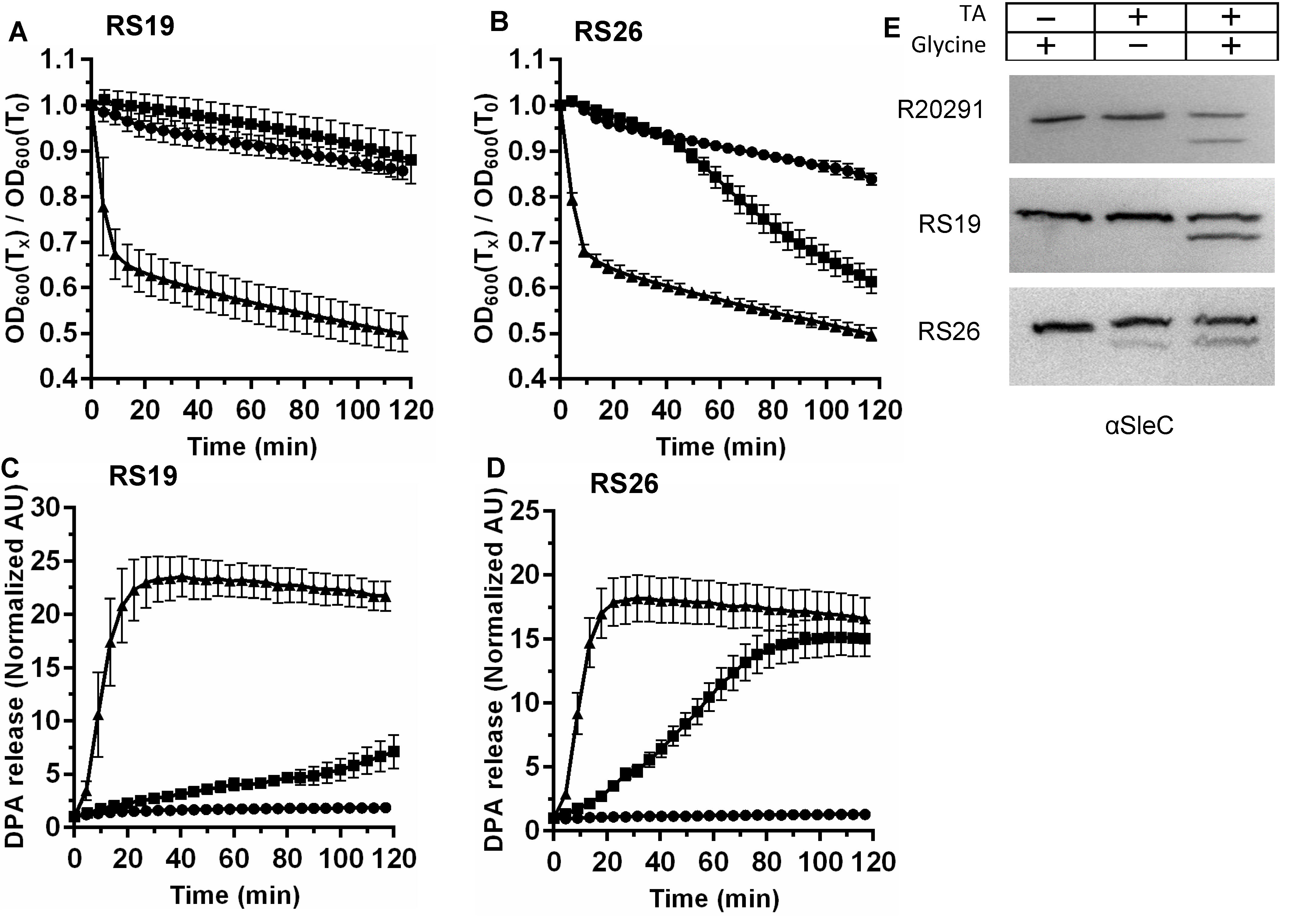

Supplement: S5 Fig — (A,B) Spores purified from C. difficile RS19 (CRG2359 with restored pyrE) and RS26 (cspBAΔ560–586; restored pyrE) strains were suspended in buffer supplemented with (black circle) 30 mM glycine or (black square) 10 mM TA or (black triangle) 10 mM TA and 30 mM glycine and OD600 was monitored over time. (C, D) CaDPA release from the indicated strains was determined as in A & B with buffer also supplemented with 250 μM Tb3+. Data points represent the averages from three technical triplicates of biological duplicate experiments and error bars represent the standard error of the mean. (E) The activation of SleC from spores purified from R20291, RS19 and RS26 in presence of buffered 30 mM glycine or 10 mM TA or both 10 mM TA and 30 mM glycine was determined after incubation for 2 hours at 37°C. (F) Equal numbers of spores derived from R20291, RS19 or RS26 were extracted, separated by SDS-PAGE and transferred to PVDF membranes for immunoblotting of CspB, CspA and CspC. (TIF) [file ppat.1007681.s005.tif]

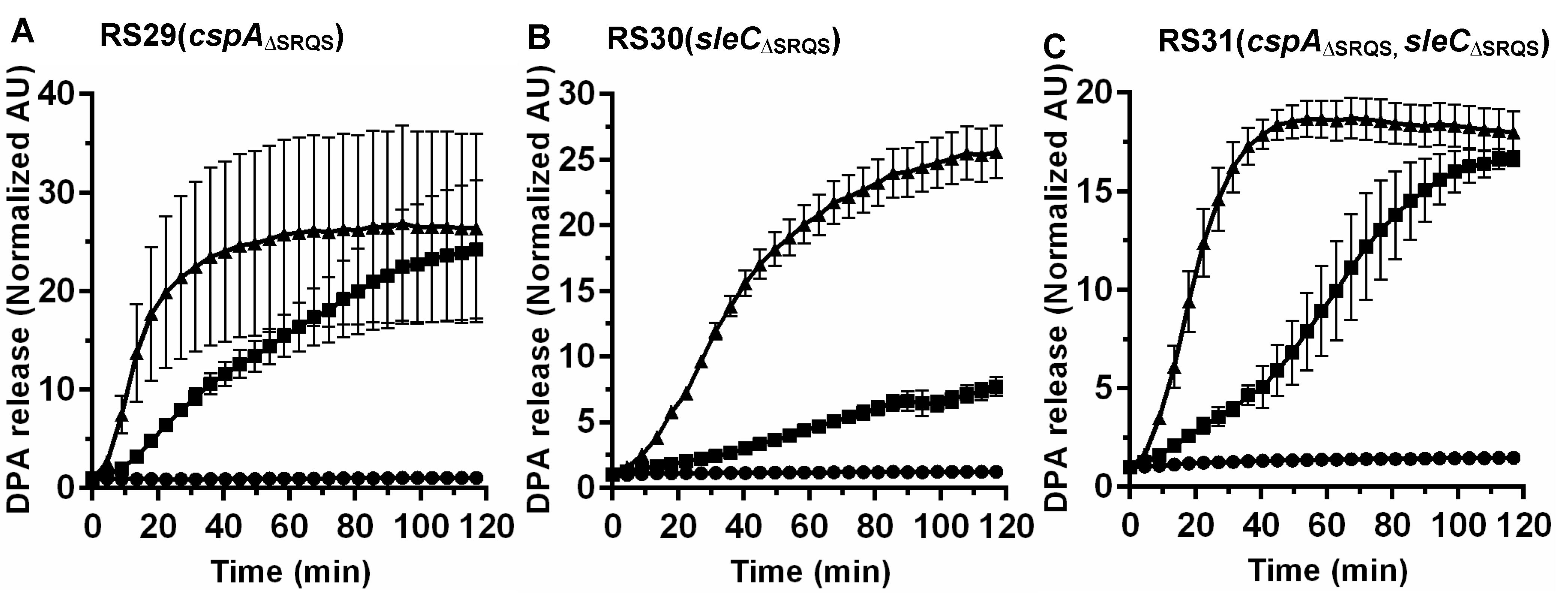

Supplement: S6 Fig — CaDPA release during spore germination of the indicated strain was monitored by suspending spores in buffer supplemented with Tb3+ and (black circle) 30 mM glycine or (black square) 10 mM TA or (black triangle) 10 mM TA and 30 mM glycine. (A) RS29 (cspAΔSRQS), (B) RS30 (sleCΔSRQS), (C) RS31 (cspAΔSRQS; sleCΔSRQS). Data points represent the averages from three technical triplicates of biological duplicate experiments and error bars represent the standard error of the mean. (TIF) [file ppat.1007681.s006.tif]
