# Supplementary material for: The requirement for co-germinants during Clostridium difficile spore germination is influenced by mutations in yabG and cspA
Source: PLoS Pathog. 2019 Apr 3;15(4):e1007681. doi: 10.1371/journal.ppat.1007681 (PMC6464247; doi:10.1371/journal.ppat.1007681)
Supplement: S1 Table — The oligonucleotides used in this study are listed. (DOCX) [file ppat.1007681.s007.docx]

**Table S1. Oligonucleotides used in this study**

| Primer # | Name of primer | Primer Sequence (5’ to 3’) | |
| --- | --- | --- | --- |
| 156 | 5' tcdB | TTACATTTTGTTTGGATTGGAGGTC | |
| 157 | 3' tcdB | AGCAGCTAAATTCCACCTTTCTACC | |
| 160 | 5' sleC | ATGCAAGATGGTTTCTTAACAGTAAGC | |
| 161 | 3' sleC | TTAAATTAAAGGATTTAAAGAAGCTATTC | |
| 173 | 5' catP 3 | ATGGTATTTGAAAAAATTGATAAAAATAG | |
| 174 | 3' catP 2 | TTAACTATTTATCAATTCCTGCAATTCG | |
| 207 | 5'Tn916ApaI | AA GGGCCC TAA CAT CTT CTA TTT TTC CCA AAT CC | |
| 208 | 3'Tn916ApaI | AA GGGCCC C960 | |
| 466 | 5'pET_SleC | ttttgtttaactttaagaaggagatatacatatgcaagatggtttcttaacagtaagc |  |
| 467 | 3'pET_SleC | atctcagtggtggtggtggtggtgctcgagaattaaaggatttaaagaagctatt |  |
| 578 | 5'pJS116_cspBA 2 | cgaattcgagctcggtacccggggatcctctagaaaaactataaagttataattgttggagatgct | |
| 813 | yabG (279s) gBlock | TTCCCCTCTAGAAAAAAGCTTATAATTATCCTTAGTTCCCAAACTTGTGCGCCCAGATAGGGTGTTAAGTCAAGTAGTTTAAGGTACTACTCTGTAAGATAACACAGAAAACAGCCAACCTAACCGAAAAGCGAAAGCTGATACGGGAACAGAGCACGGTTGGAAAGCGATGAGTTACCTAAAGACAATCGGGTACGACTGAGTCGCAATGTTAATCAGATATAAGGTATAAGTTGTGTTTACTGAACGCAAGTTTCTAATTTCGATTGGAACTCGATAGAGGAAAGTGTCTGAAACCTCTAGTACAAAGAAAGGTAAGTTATGAAGTTTGACTTATCTGTTATCACCACATTTGTACAATCTG | |
| 906 | 5' XbaI_Prom_YabG | GATCCTCTAGAGAAATGTTTTTTTGACATTAG | |
| 907 | 3' YabG_XhoI | GCCTCGAGCTAATGTAATATTGTTTTTGGC | |
| 924 | 5' pJS116_XbaI_YAbG | TTCGAGCTCGGTACCCGGGGATCCTCTAGAGAAATGTTTTTTTGACATTAGG | |
| 925 | 3' YabG_XhoI_pJS116 | GTGCCAAGCTTGCATGTCTGCAGGCCTCGAGCTAATGTAATATTGTTTTTGGC | |
| 943 | 5'cspB_UP | AATTTTTTTATCAGGAAACAGCTATGACCGCGGCCGCTAATGGTGGTAATTATTGGATAG | |
| 1056 | 3' CspC_pJS116 | CAAGCTTGCATGTCTGCAGGCCTCGAGTTATCTATAGAGTATTTGCTATCTGTTGAAT | |
| 1084 | 5' pyrE 2 | GTCCAGTGTTCTGGGGAG | |
| 1085 | 3 'pyrE 2 | AAAATTTACATTTTTTAAGTAACACTATAAATAATTAAGTTTTTA | |
| 1086 | 3'cspBAC_UP | AATCAATTATAATTTTACATAGGTTCTTATCTCGACTTCTAAAATTATTATTAAT | |
| 1087 | 5'cspBAC_DN | TATACATTAATAATAATTTTAGAAGTCGAGATAAGAACCTATGTAAAATTATAATTG | |
| 1088 | 3'cspBAC_DN | TGGGTCTGCGATCGCGCATGTCTGCAGGCCTCGAGTCTTTTACTGTTATAAATTCCTTTT | |
| 1160 | 5' YN4_1Kb UP_CspBdelA | TTCGAGCTCGGTACCCGGGGATCCTCTAGAAAATATAAGTTATGGAAGTAATGAA | |
| 1163 | 3' 1Kb DN_cspBdelA_YN4 | TGCCAAGCTTGCATGTCTGCAGGCCTCGAGCCAAAGTTCTAATGATAATTCTT | |
| 1232 | 3' 1kb UP cspBA_75nt | CTTATATAAATTTCATTCTCTAAGTCATTATATCCAATATCCTCCGTT | |
| 1233 | 5' 1Kb DN cspBA_75nt | AAGATTTAGAAACGGAGGATATTGGATATAATGACTTAGAGAATGAAATTTAT | |
| 1234 | 3' 1Kb UP_cspBA_102nt | ACTTGCATTTTTACTTATATAAATTTCATTCGTTTCTAAATCTTGATTTATACTA | |
| 1235 | 5' 1Kb DN_cspBA_102nt | CTATCTAGTATAAATCAAGATTTAGAAACGAATGAAATTTATATAAGTAAAAATGC | |
| 1236 | 3' 1Kb UP_cspA_78nt | GGGAGTATGGACTACATCTATTCCTGATAGATCTATAAACTTATACCTATTTTCCTC | |
| 1237 | 5' 1Kb DN_cspA_78nt | GATGAGGAAAATAGGTATAAGTTTATAGATCTATCAGGAATAGATGTAGTCCA | |
| 1238 | 3' 1Kb UP_cspB_78nt | AAACTTATACCTATTTTCCTCATCTTTAAAATCTGATAAATTTAAAAAACCAA | |
| 1239 | 5' 1Kb DN_cspB_78nt | TCAGGATTTGGTTTTTTAAATTTATCAGATTTTAAAGATGAGGAAAATAGGT | |
| 1258 | 3' cspB_A _75aa_pRS114 | TTTCATTCTCTAAGTCATTATA | |
| 1259 | 3' cspB_A _102aa_pRS115 | ACTTATATAAATTTCATTCGT | |
| 1260 | 3' cspA _76aa_pRS116 | TATTCCTGATAGATCTA | |
| 1261 | 3' cspB_76aa_pRS117 | CCTCATCTTTAAAATCTGAT | |
| 1287 | 3' 1Kb UP CspA_150nt | TACACCTAATGAATCACTTATCTTAAAAAAATCTATAAACTTATACCTATTTTCCT | |
| 1288 | 5' 1Kb DN CspA_150nt | GATGAGGAAAATAGGTATAAGTTTATAGATTTTTTTAAGATAAGTGATTCATTAG | |
| 1361 | 5' YN4_SleC_1kB UP | TTCGAGCTCGGTACCCGGGGATCCTCTAGAAGCTAAAAATACAGAGTAAATAATAC | |
| 1362 | 3' SleC_SQRS Del_1Kb UP | AGTATGTTCTCCTATGTCAAAAATCAACTCGAAAGAACGCTTACTTCTTG | |
| 1363 | 5' SleC_SRQS del_1kB DN | CTCCTAGAACAAGAAGTAAGCGTTCTTTCGAGTTGATTTTTGACATAG | |
| 1364 | 3' YN4_SleC_1Kb DN | TGCCAAGCTTGCATGTCTGCAGGCCTCGAGATAATAAATAATAATTTAGATAA | |
| 1365 | 3' SRQS del cspA | ATTCCTGATTGTCTACT | |
| 1376 | 5' SleC SRQS del | GCGTTCTTTCTCAAGACAAAGT | |
| 1466 | pet22b_YabG_Fp | ttttgtttaactttaagaaggagatatacaatgaaggtaggagacattgtag | |
| 1467 | pet22b_YabG_Rp | caagcttgtcgacggagctcgaattcggatcctactagtggtggtggtggtggtgatgtaatattgtttttggcat | |
| 1572 | YABG_slec_Rp | ccatcttgcatatgtatatctccttcttatcactaatgtaatattgtttttggc | |
| 1573 | yabg_SLEC_Fp | attacattagtgataagaaggagatatacatatgcaagatggtttcttaac | |
| 1680 | pet22b_yabg_**SleC6his**_Rp | agctcgaattcggatcctactagtggtggtggtggtggtgaattaaaggatttaaagaagctat | |
| 1681 | pet22b_SleC_Fp | ttttgtttaactttaagaaggagatatacatatgcaagatggtttcttaacag | |
| 1682 | Pet22b_SleC_QSELI DEL_Rp | cctatgtcaaatcttgagaaagaacgcttacttcttgttctaggagacatgggaac | |
| 1683 | Pet22b_SleC_QSELI DEL_Fp | taagcgttctttctcaagatttgacataggagaacatactctatatggtacttatc | |
